# Biodiversity and genetic improvement of *Moringa oleifera*: traditional breeding and genome editing strategies for reducing anti-nutritional factors

**DOI:** 10.3389/fpls.2026.1869631

**Published:** 2026-09-07

**Authors:** Shamini Karunagaran, Saraladevi Muthusamy, Anandhavalli Manikandan, Prabhakaran Radhamma Geetha Lekshmi, Subramaniam Leelavathi, Praveen Gidagiri, Senjaeriputhur Devaraj Sivakumar, Suganya Kanna Selvaraj, Vangili Ramasamy Saminathan, Chakkarapani Babu, Kalipatty Nalliappan Ganesan, Balaji Kannan, Ramamoorthy Pushpam, Sudhagar Rajaprakasam, Selvaraju Kanagarajan

**Affiliations:** 1 Genetics and Plant Breeding, Tamil Nadu Agricultural University, Coimbatore, India; 2 Department of Plant Breeding, Swedish University of Agricultural Sciences, Lomma, Sweden; 3 Department of Postharvest Management, College of Agriculture, Kerala Agricultural University, Vellayani, Thiruvananthapuram, India; 4 Department of Agronomy, Tamil Nadu Agricultural University, Coimbatore, India; 5 Department of Entomology, Tamil Nadu Agricultural University, Coimbatore, India; 6 Soil and Water Conservation Engineering, Tamil Nadu Agricultural University, Coimbatore, India; 7 School of Science and Technology, The Life Science Centre, Örebro University, Örebro, Sweden

**Keywords:** anti-nutritional factors, breeding, genetic diversity, microbial fermentation, moringa, omics approach, transgene-free genome editing

## Abstract

Rapid population growth demands research on alternative, nutritionally rich food crops that can sustain food security, mitigate cultivation risks associated with climate change, and reduce dependence on mono- and few-crop systems. Several underutilized crops have been cultivated for centuries, but their use often remains country- or continent-specific, with limited scientific documentation and dissemination to regions facing major nutritional challenges. Moringa is one such promising crop; with rich nutrients, its plant parts, from root to pod, are edible or useful, and it also contributes to environmental cleansing. However, its wider utilization is constrained by anti-nutritional factors (ANFs) such as alkaloids, tannins, saponins, oxalates, glucosinolates (GSLs) and phytic acid, which reduce nutrient bioavailability and may cause adverse health effects. Various methods, including microbial fermentation, have proven effective at reducing these ANFs. For instance, lactic acid and solid-state fermentation can substantially reduce the enzymatic degradability of ANFs such as phytate, tannins, and GSLs while improving mineral bioavailability. In parallel, genetic and biotechnological approaches improving moringa offer substantial potential to alleviate malnutrition and enhance environmental and agronomic performance through better yield, quality, and stress resilience, along with ANF reduction. This review synthesizes current knowledge on moringa botany, crop diversity and summarizes genetic and biotechnological approaches, including omics resources, conventional breeding, microbial fermentation, and emerging genome-editing strategies, to improve yield and nutritional quality while reducing ANFs. By outlining the benefits of moringa, identifying major knowledge gaps, and highlighting future research priorities, this review aims to guide the strategic development and global deployment of moringa as a resilient, nutrient-dense crop for sustainable food and nutrition security.

## Introduction

Global population growth, climate change, and over-reliance on a few staple cereals threaten future food and nutrition security. Diversifying production with nutrient-dense, climate-resilient crops is therefore a key strategy to reduce vulnerability to yield shocks and micronutrient deficiencies.

*Moringa oleifera* is often described as a multipurpose or “miracle” tree because almost every part of the plant is used as food, feed or raw material: young pods and leaves as vegetables; dried leaf powder as a micronutrient supplement; seed cake as livestock feed, roots, bark, and flowers in traditional medicine; and seed oil for cosmetics and biodiesel (Gopalakrishnan et al., 2016; Mabusela et al., 2018; Karunagaran et al., 2025). It provides numerous health benefits, including anti-cancer, anti-inflammatory, anti-diabetic, and anti-bacterial properties (Ma et al., 2020). Also, the bioactive compounds (polyphenols and alkaloids) in moringa help reduce the risks of chronic diseases (Vergara-Jimenez et al., 2017). In addition, moringa is used in agroforestry systems as a living fence and shade tree, and crushed seeds serve as natural coagulants for water clarification, giving the crop relevance for both nutritional and environmental benefits (Camacho et al., 2017; Coriolano et al., 2019; Devkota and Bhusal, 2020; Desta and Bote, 2021; Yamaguchi et al., 2021; Horn et al., 2022).

**Figure 1:**
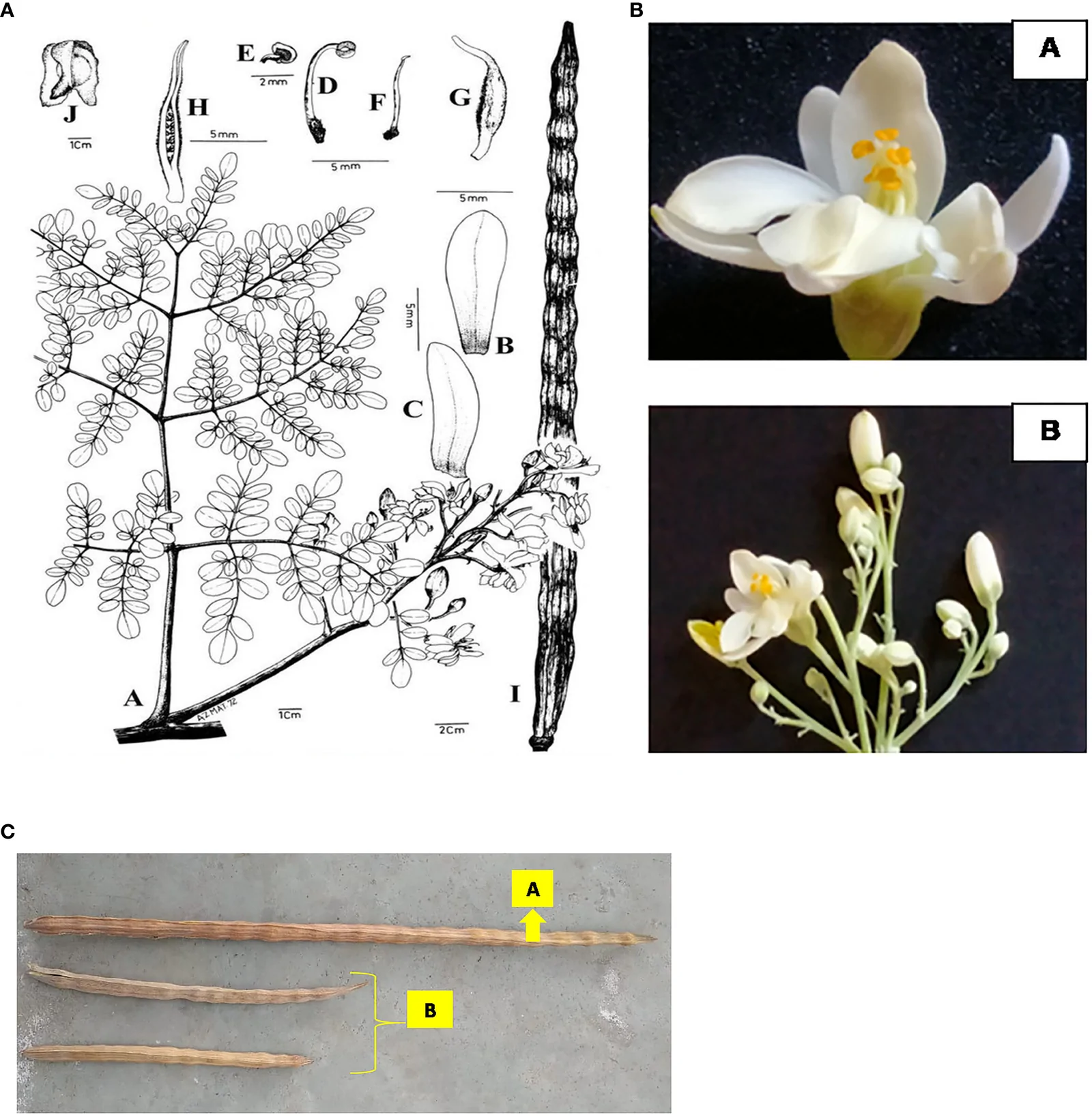
**(A)** Dissection image of *M. oleifera* flower A, Branch; B, Petal; C, Sepal; D, Fertile stamen; E, Anthers; F, Sterile stamen; G, Carpel; H, L.S. of ovary; I, Fruit; J, Seed. (Source: https://www.worldfloraonline.org). **(B)** Flower of *M. oleifera*: A, Individual flower of *M. oleifera*; B, Inflorescence of *M. oleifera*. **(C)** Diversity in pod length of *M. oleifera*: A, Annual moringa; B, Perennial moringa.

*M. oleifera* is a diploid species (2n = 28) belonging to the family *Moringaceae*. Moringa has a deep root system with spreading lateral roots. **Figure 1A** illustrates the morphology of *Moringa oleifera*. The leaves are compound, consisting of eight to ten pairs of pinnae, each bearing two pairs of dark green leaflets with a rounded apex and red-tinged veins. **Figure 1B** shows the floral morphology of *M. oleifera* at two scales: an individual flower (A) and the inflorescence (B). The flowers are hermaphroditic, zygomorphic, pentamerous, and white or creamy white; as seen in the individual flower (A), the calyx consists of five linear, lobed sepals that are reflexed, lanceolate, and tubular outside, and the corolla bears five petals and stamens surrounding a superior ovary. The inflorescence (B) is a axillary panicle; moringa flowers twice yearly, or year-round in some varieties, and is predominantly cross-pollinated, a reproductive trait that shapes the breeding strategies discussed below. **Figure 1C** illustrates the diversity in pod length in moringa, comparing the pod of an annual type (A) with those of a perennial type (B). The fruit is a three-valved, elongated, non-dehiscent pod whose length varies from 30 to 120 cm and contains dark brown to black seeds. The seeds are round with three papery wings (Anzano et al., 2021).

*Moringa* species are fast-growing, drought-tolerant trees and shrubs distributed primarily across the Indian subcontinent, East Africa, and the Arabian Peninsula, with *Moringa oleifera* Lam. being the most widely cultivated species (Olson and Carlquist, 2001). The name “moringa” is derived from the Tamil word “murungai” and reflects its long history of cultivation in South India (Abdelsalam and Fathi, 2023). Although widely grown throughout the tropics and subtropics, *M. oleifera* is believed to have originated in the sub-Himalayan region of Northern India (Leone et al., 2015).

**Figure 2:**
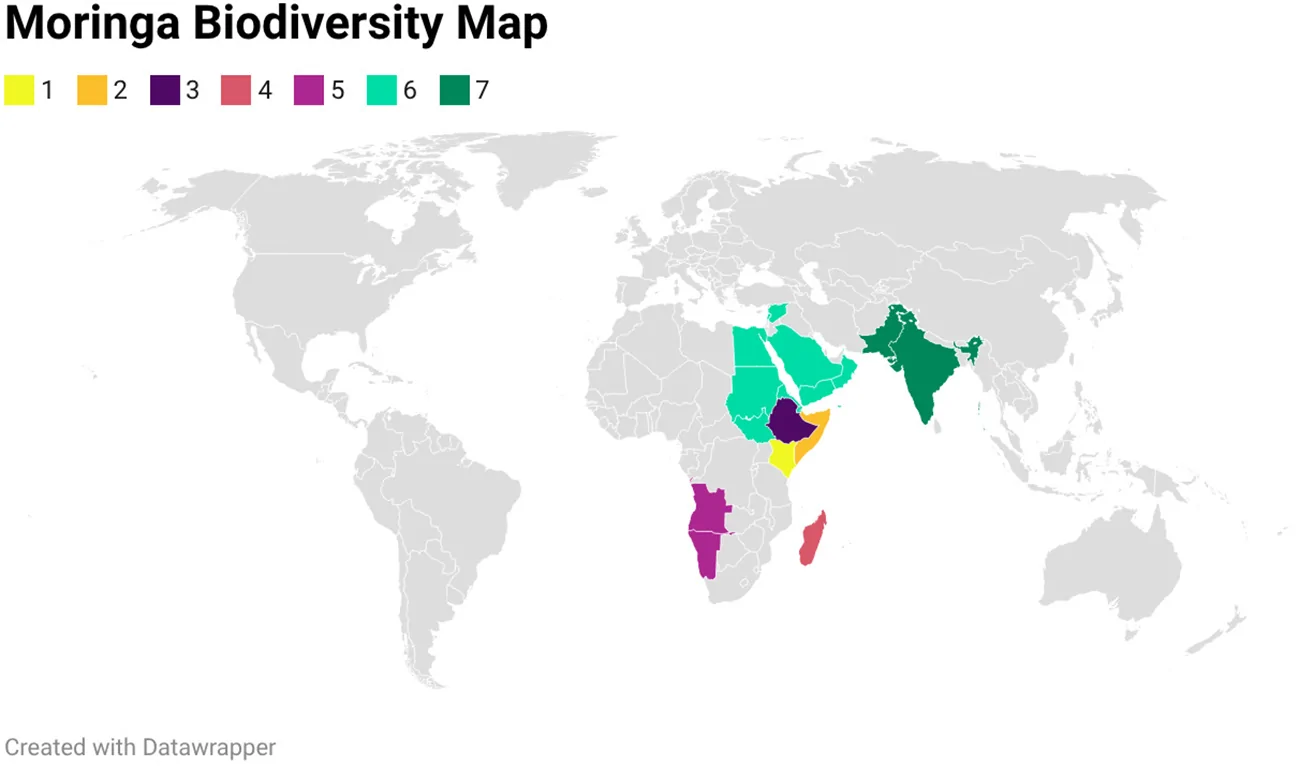
Global distribution *Moringa species*: 1. *M. arborea* Verdc., *M. stenopetala* (Baker f.) Cufod., *M. longituba* Engl., *M. rivae* Chiov., *M. borziana* Mattei 2. *M. borziana* Mattei, *M. longituba* Engl., *M. pygmaea* Verdc. 3. *M. ruspoliana* Engl., *M. stenopetala* (Baker f.) Cufod., *M. longituba* Engl., *M. rivae* Chiov., *M. borziana* Mattei 4. *M. drouhardii* Jum., *M. hildebrandtii* Engl. 5. *M. ovalifolia* Dinter & A. Berger 6. *M. peregrina* (Forssk.) Fiori 7. *M. concanensis* Nimmo ex Dalzell & A. Gibson and *M. oleifera* Lam.

Moringa thrives in both arid and semi-arid environments. The genus *Moringa* has 13 cultivable species: 1. *M. arborea* Verdc.; 2. *M. borziana* Mattei; 3. *M. concanensis* Nimmo ex Dalzell & A. Gibson.; 4. *M. drouhardii* Jum.; 5. *M. hildebrandtii* Engl.; 6. *M. longituba* Engl.; 7. *M. oleifera* Lam.; 8. *M. ovalifolia* Dinter & A. Berger; 9. *M. peregrina* (Forssk.) Fiori.; 10. *M. pygmaea* Verdc.; 11. *M. ruspoliana* Engl.; 12. *M. rivae* Chiov.; and 13. *M. stenopetala* (Baker f.) Cufod (Leone et al., 2015; Mashamaite et al., 2020). **Figure 2** depicts the geographical distribution of *Moringa* species. Although the genus as a whole spans Africa and Asia, species richness is heavily concentrated in Africa, with only a few species native to Asia; the country-wise distribution is summarized in **Table 1**. Beyond cataloging occurrence, this pattern reveals a strong center of species diversity in the Horn of Africa and adjacent arid zones, where most of the 13 species are endemic, in contrast to the near-global but largely single-species (*M. oleifera*) cultivated distribution elsewhere, a contrast with direct implications for where wild genetic resources for breeding must be sourced and conserved.

**Table 1:** Global distribution of *Moringa* species.

Moringa species- label	Moringa species	Countries	References
1	*Moringa arborea* Verdc., *Moringa stenopetala* (Baker f.) Cufod., *Moringa longituba* Engl., *Moringa rivae* Chiov., *Moringa borziana* Mattei	Kenya	Plants of the World Online, 2026 (POWO, 2026) (Data available at https://powo.science.kew.org/) Leone et al., 2015; Mashamaite et al., 2020 and Boopathi et al., 2021.
2	*Moringa borziana* Mattei, *Moringa longituba* Engl., *Moringa pygmaea* Verdc.	Somalia	
3	*Moringa ruspoliana* Engl., *Moringa stenopetala* (Baker f.) Cufod., *Moringa longituba* Engl., *Moringa rivae* Chiov., *Moringa borziana* Mattei	Ethiopia	
4	*Moringa drouhardii* Jum., *Moringa hildebrandtii* Engl.	Madagascar	
5	*Moringa ovalifolia* Dinter & A. Berger	Namibia, Angola	
6	*Moringa peregrina* (Forssk.) Fiori	Djibouti, Egypt, Eritrea, Ethiopia, Gulf States, Lebanon-Syria, Oman, Palestine, Saudi Arabia, Sinai, Somalia, Sudan-South Sudan, and Yemen	
7	*Moringa concanensis* Nimmo ex Dalzell & A. Gibson *Moringa oleifera* Lam.	India and Pakistan	

**Figure 3:**
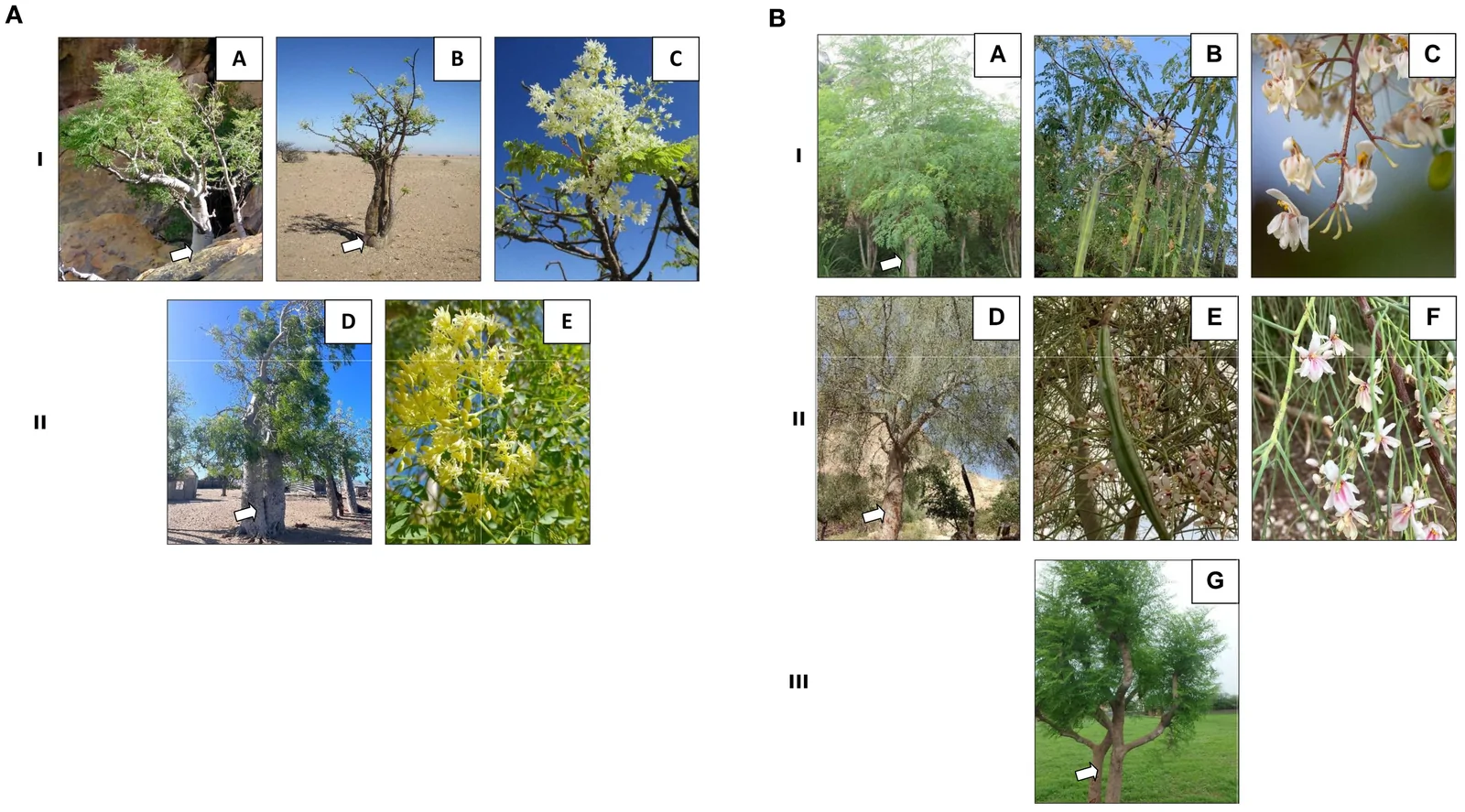
**(A)** Bottle-trunked *Moringa* species: I **(A–C)**, *M. ovalifolia* Dinter & A. Berger, II **(D, E)**, *M. drouhardii* Jum. Arrow indicates bottle trunk. (Source: https://www.worldfloraonline.org). **(B)** Slender-trunked Moringa species: I **(A–C)**, *M. oleifera* Lam.; II **(D–F)**, *M. peregrina* (Forssk.) Fiori; III **(G)**, *M. concanensis* Nimmo ex Dalzell & A. Gibson. Arrow indicates slender trunk (Source: https://www.worldfloraonline.org).

These *Moringa* species are further classified into four morphological groups (Olson and Carlquist, 2001). (1) Bottle trunk trees: This group includes *M. ovalifolia*, *M. stenopetala* (Baker f.) Cufod., *M. drouhardii* Jum., and *M. hildebrandtii* Engl. **Figure 3A** represents the bottle-trunk types (bottle-like stems with radially symmetrical flowers). Distinctively, in this group, *M. drouhardii* Jum. and *M. hildebrandtii* Engl. have branches and leaves only at the top of the trees and give a palm-like appearance. (2) Sarcorhizal trees: This group includes 2 species, *M. arborea* Verdc.*, M. ruspoliana* Engl. (3) Slender trunk trees: **Figure 3B** describes the slender trunk trees. This group includes three species, *M. concanensis* Nimmo, *M. oleifera* Lam., *M. peregrina* (Forssk.) Fiori. (4) Tuberous shrubs: This group comprises four species, *M. borziana* Mattei, *M. longituba* Engl., *M. pygmaea* Verdc., and *M. rivae* Chiov. These species are shrubs and herbs with tubers. Among these *M. arborea, M. borziana, M. longituba, M. rivae, M. ruspoliana, and M. stenopetala* are categorized as endangered, with *M. arborea* specifically listed as a threatened species (World Conservation Monitoring Centre, 1998), while *M. hildebrandtii* has been classified as extinct (Olson and Razafimandimbison, 2000).

Numerous reviews have summarized the phytochemistry, pharmacology, and nutritional properties of *M. oleifera* and its applications as a functional food or medicinal plant (Azlan et al., 2022; Kashyap et al., 2022; Pop et al., 2022; Pareek et al., 2023). However, previous work has paid comparatively little attention to the genetic resources and breeding history of moringa, emerging omics and genome resources, and the systematic comparison of biological ANFs-reduction strategies, such as microbial fermentation versus new genomic techniques (NGTs), including genome editing. This review therefore (i) synthesizes current knowledge on crop improvement approaches, (ii) summarizes available omics data and candidate genes related to major ANFs, and (iii) critically evaluates microbial fermentation and genome-editing-based strategies for reducing ANFs, highlighting key research gaps and future directions for developing low-ANF, high-nutrient moringa cultivars and products.

## Crop improvement approaches

Moringa crop improvement is critical for unlocking its underutilized nutritional and medicinal potential and for making it a reliable contributor to global food security. The existence of substantial genetic variability across globally maintained germplasm collections constitutes the prerequisite for any breeding program. The Indian Council of Agricultural Research - National Bureau of Plant Genetic Resources (ICAR-NBPGR), India holds 257 accessions; the Horticultural College and Research Institute (HC&RI) of Tamil Nadu Agricultural University (TNAU), Periyakulam, Tamil Nadu, India maintains approximately 70–90 accessions; and the World Vegetable Centre, Taiwan collection spans more than 70 accessions from 12 countries across Africa, Asia, and the America and Moringa Genebank of Embrapa Tabuleiros Costeiros, Sergipe state, Brazil holds 25 accessions (Alavilli et al., 2022; Soares et al., 2024).

In India, moringa trees are commonly grown in backyards as multipurpose trees, particularly in South Indian states such as Tamil Nadu, Karnataka, Kerala, and Andhra Pradesh.Varied annual and perennial genotypes are grown in India, particularly Tamil Nadu, depending on local production goals, market demand, and food habits; the principal ecotypes, Moolanur moringa, Karumbu murungai, Chavakacheri murungai, Jaffna moringa, and Kappalpatti murungai, are each characterized by distinct pod size, flesh content, plant stature, flowering, and color (**Table 2**). Read together, these ecotypes show that much of moringa’s exploitable variation in pod and stature traits already resides in farmer-maintained perennial germplasm rather than in formally bred lines, underscoring the value of characterizing this material systematically before it is lost.

**Table 2:** Genetic resources (local perennial moringa accessions) of annual and perennial moringa (*M. oleifera* and *M. concanensis*) in Tamil Nadu, India (Boopathi et al., 2021; Kumar et al., 2014).

S. No.	Accession	District(s)	Particulars
1.	Moolanur moringa	Karur and Dharapuram	• Pods are typically 45-50 cm in length; produce 200 kg/tree • 15-year lifespan without pruning
2.	Valayapatti moringa	Madurai	• It has a pod length and weight of 65 cm and 120 g, respectively • The average number of pods per tree per harvest is 1000-1200
3.	Chavakacheri moringa	All districts	• It was introduced to India from Sri Lanka • The pod length is 90-120 cm and produces 500-600 pods/tree/year • The extra pod length poses marketing issues due to the chances of transport- related damage
4.	Chemmurungai	All districts	• The cultivation advantages include year-round pod production and high-yield • It has red-tinged midrib and pod tips
5.	Jaffna	Tirunelveli and Tuticorin	• It is a native accession of Yazphanam, Sri Lanka • It is a good yielder, pods are 60-90 cm long, and has good taste
6.	Kattumurungai or Peyimurungai	Perambalur	• It comes under the species *Moringa concanensis* • Cultivated in marginal lands with poor soil characteristics and produces lengthy pods, which is the reason for its name “Peyimurungai” (meaning lengthy pods)
7.	Kodikkalmurungai	Tiruchirapalli	• This accession is planted in wetland or semi-wetland conditions with a view to providing support to betel vine climbers • It has multi-utility and good taste. It produces 20-25 cm lengthened pods
8.	Palmurungai	Kanyakumari	• It tastes good and has a thick pulp • The average pod yield count/tree/season is 100
9.	Punamurungai	Tirunelveli and Kanyakumari	• It produces good biomass and medium-length pods, which makes it suitable for ideal home garden situations
10.	Palamedu moringa	Madurai	• The pod length is 60 cm, and the average pod weight is 95-100 g. It yields 100 pods in a season/tree

Together, these germplasm collections will constitute a valuable reservoir of allelic diversity that can be systematically exploited to address key breeding objectives.

These key breeding objectives would include: (1) Yield improvement: Developing cultivars with increased leaf biomass and pod yield; (2) Nutritional biofortification: Developing cultivars with elevated concentrations of iron, zinc, protein, β-carotene, and vitamins; (3) Early maturity: Short-duration, annual cultivars that initiate early flowering and pod set; (4) Off-season cultivars: Year-round pod harvest in perennial moringa cultivars; (5) Disease and pest resistance: Develop cultivars with increased resistance to common pathogens (powdery mildew, root rot) and pests (bark borer, pod fly) (6) Abiotic stress tolerance: Develop varieties tolerant to extreme climatic conditions (heat, drought, salinity). (7) Mechanized harvesting: Develop short-statured and annual-type plants with uniform flowering amenable to mechanized harvest. (8) Extended shelf-life: Extended post-harvest shelf life of leaves and pods. Collectively, these objectives define a comprehensive breeding framework for transforming moringa into a resilient, nutritionally dense, and commercially viable crop.

Extensive progress has been made in breeding annual moringa, especially in southern India, leading to the release of several high-yielding varieties by the Indian Institute of Horticultural Research (IIHR) and state agricultural universities (Varalakshmi and Devaraju, 2007). Plant introduction was one of the earliest approaches employed: Sri Lankan genotypes such as ‘Jaffna’ and ‘Chavakacheri murungai’ were evaluated and successfully introduced into Indian production systems, demonstrating adaptation to South Indian agroclimatic conditions and contributing to local germplasm diversity (Minakshi et al., 2021). Mass selection and classical hybridization are the main conventional breeding approaches used for moringa improvement. Mass selection involves selecting visually similar plants, allowing open pollination, and bulk-collecting equal seed quantities from them; this method helped develop the annual cultivar PKM 1 at Tamil Nadu Agricultural University, Coimbatore. PKM 1 was developed primarily through mass selection, with subsequent purification achieved via pure-line selection. In contrast, classical hybridization combines genetically distinct parents to create improved progeny, as seen in PKM 2, which was developed from a cross between MP 31 and MP 38 and showed a higher yield than PKM 1.

Similarly, mutation breeding relies on inducing heritable genetic changes using physical mutagens, such as gamma rays, or chemical mutagens, such as ethyl methane sulphonate (EMS) and methyl methane sulphonate (MMS), to generate novel variation for selection. From the annual moringa variety PKM 1, several mutant genotypes, *viz*., 15-1-09, 35-1-62, 35-1-63, and 35-1-68, were developed through gamma rays (100–300 Gy) and EMS (0.15-0.25%). Among these, superior mutants 15-1-09-39 and 35-1-62-72 exhibited high diversity for hypocotyl and shoot color, leaf biomass, pod yield, and growth habit, indicating the effectiveness of induced mutagenesis for improving yield-related and morphological traits in moringa (Bharathi et al., 2024).

A recurring challenge emerges from conventional breeding approaches in moringa. Mass selection has successfully produced released cultivars, most notably PKM 1, but repeated selection may reduce the genetic variation available for continued genetic improvement. Conversely, approaches that preserve or generate new variation, such as hybridization and induced mutagenesis, face practical challenges because moringa is an obligate outcrossing species, making the development and maintenance of stable inbred parental lines difficult. Collectively, these factors may contribute to the relatively limited number of released cultivars, which have primarily been selected for agronomic performance, particularly yield, rather than for reduced ANFs. Consequently, while conventional breeding remains effective for improving many agronomic traits, it appears less well suited for the complex, quantitative, and biochemically regulated ANF traits emphasized in this review. This limitation highlights the potential value of integrating conventional breeding with omics-enabled gene discovery and genome-editing approaches to accelerate the development of nutritionally improved moringa cultivars.

Molecular characterization of natural populations in moringa has been done using Amplified Fragment Length Polymorphism (AFLP) and Random Amplified Polymorphic DNA (RAPD) markers (Muluvi et al., 1999; Saini et al., 2013; Rufai et al., 2013). A co-dominant Simple Sequence Repeat (SSR) marker was identified and used, along with morphological markers, for diversity studies, thereby enhancing efficiency in moringa breeding programs (Minakshi et al., 2021). The genetic stability of various micropropagation systems can be ensured by using Random Amplified Microsatellite Polymorphism (RAMP) markers (Avila-Trevino et al., 2017), providing a quality-control tool for elite genotype multiplication. Kumar et al. (2017) reported that RAPD and cytochrome P450 markers (CytP450) produced substantial polymorphism, with 79.68% and 86.44% polymorphic bands, respectively. These high polymorphism values make RAPD and CytP450 markers effectively useful to identify allelic variants in closely related moringa genotypes for breeding purposes. A 2024 study at ICAR-Indian Institute of Horticultural Research (Bangalore) found that several Expressed Sequence Tag - Simple Sequence Repeat (EST-SSR) markers had sequence similarity to PR (pathogenesis-related) genes, suggesting direct utility for screening disease resistance in breeding programs (Narayana et al., 2024).

Taken together, these marker studies indicate that moringa harbors substantial exploitable genetic variation, although the available evidence remains methodologically fragmented and difficult to integrate across studies. Early RAPD and AFLP analyses (Muluvi et al., 1999; Saini et al., 2013; Rufai et al., 2013) established the presence of considerable genetic diversity but relied on dominant marker systems, limiting cross-study comparability. More recent co-dominant SSR and EST-SSR markers (Minakshi et al., 2021; Narayana et al., 2024) provide more transferable and potentially function-linked tools for breeding, although their application has so far been limited to relatively small and geographically restricted germplasm panels. Consequently, while molecular characterization has advanced considerably over the past three decades, the current literature does not yet indicate a widely adopted core germplasm collection or trait-linked marker set for breeding applications. Future efforts should therefore prioritize integrating existing germplasm resources into a shared, genotyped and phenotyped reference panel that links molecular variation with ANFs and nutritional traits, thereby enabling more effective marker-assisted selection and genomic improvement.

Beyond marker-assisted selection, genome-wide association studies (GWAS) and genomic selection have transformed the improvement of quality and anti-nutritional traits in major crops, including the dissection of GSL variation in *Brassica* and rapid-cycle selection in cereals (Dreisigacker et al., 2023; Kittipol et al., 2019; Tan et al., 2021). In moringa, however, these approaches are not yet practically deployable because the necessary large, structured, and densely genotyped reference population with matched phenotyping for ANF and nutritional traits is lacking. Developing such a panel across the available germplasm collections is therefore a prerequisite for future GWAS and genomic selection in moringa.

Some of the successful moringa varieties released by state agricultural universities in South India include PKM 1 and PKM 2 (Tamil Nadu Agricultural University), Anupama (Kerala Agricultural University), and Dhanraj (University of Agricultural Sciences, Karnataka). These annual moringa varieties form a crucial component of the seed chain. The key features of these annual moringa varieties, such as plant stature, earliness, pod length, yield potential, and suitability for intensive cultivation, are shown in **Table 3**. A notable trend across these released varieties is their convergence toward short-statured, early, annual types optimized for intensive cultivation, which have advanced yield and harvest convenience but have left nutritional quality and ANF content largely unaddressed, a gap that motivates the biofortification and ANF-reduction objectives developed in this review.

**Table 3:** Improved annual *M. oleifera* varieties predominantly cultivated in India (Boopathi et al., 2021).

S. No.	Variety	Institute	Breeding approach	Special traits
1.	PKM 1	TNAU, Tamil Nadu, India	Selection	• The fruits are fleshy and tasty. The average yield potential is 50 to 54 tonnes/ha • Ratoon is possible and maintained for 3-4 years • PKM 1 can also be grown as an intercrop in the early stages of coconut fields
2.	PKM 2	TNAU, Tamil Nadu, India	Hybridization and selection	• Has more lateral branching, produces more biomass, and bears fruits at lower heights • The average yield potential is 98 tonnes/ha/year • Can be grown as an intercrop in the typical tropical orchards
3.	KM 1	TNAU, Tamil Nadu, India	Selection	• It is a bushy type and produces pods after six months of sowing • The length of the pod is 32-37 cm and produces 226-328 pods per plant in a year • Ratoon can be maintained for three years
4.	Dhanraj	UAS, Karnataka, India	Selection	• It is an annual dwarf moringa that propagates through seeds • Flowers three months after planting and can grow up to six meters • Matured pods can be harvested from 160-170 days
5.	Anupama	KAU, Kerala, India	Selection	• It has an extended flowering period, and the average pod length is 55.5 cm, with a pod yield of 10-15 kg per tree per year

The crop improvement approach in moringa is largely driven by its germplasm diversity, which provides a strong foundation for systematic breeding programs. In India, the major species include *M. oleifera* and *M. concanensis*. Both annual and perennial types contribute valuable traits for crop improvement, such as higher yield, nutritional enhancement, early maturity, stress resistance, and improved post-harvest and mechanization traits. Conventional breeding methods were widely used for varietal development. However, the success of molecular markers and associated omics technologies in other crops is to be experimented with in moringa to hasten its crop improvement programs.

## Omics in moringa

The omics approaches, including genomics, transcriptomics, proteomics, and metabolomics for moringa, are still at an early stage but are advancing rapidly. Early metabolomics work by Bennett et al. (2003) used Liquid Chromatography-Mass Spectrometry (LC-MS) to generate the first tissue-wise profiles of GSLs and phenolic compounds across moringa organs, establishing a biochemical and metabolic foundation that can be later used for comparative and functional studies. Ndou et al. (2023) combined LC-MS with GNPS molecular networking to show that moringa leaves harbor a much more diverse flavonoid profile than previously recognized, with extensive glycosylation of quercetin, kaempferol, isorhamnetin, apigenin, luteolin, and chrysin generating numerous distinct structural isomers.

Genomic resources have advanced substantially over the last decade. The African Orphan Crop Consortium (AOCC) is currently encouraging genomic research on 101 orphan indigenous species and exotic plants from Africa, including moringa, to generate genomic data and identify the functions of the genes identified to enable crop improvement (Chang et al., 2019). An initial AOCC draft genome based on Illumina short-read sequencing predicted approximately 18,451 protein-coding genes and provided the first genome-scale framework for moringa improvement (Chang et al., 2019). This was superseded by a chromosome-scale assembly produced by Chang et al. (2022) using Oxford Nanopore long reads and Hi-C scaffolding, which resolved 22,714 annotated genes. The assembly revealed that tandem duplications play an important role in the biosynthesis of secondary metabolites, including GSLs, flavonoids, and alkaloids. Complementarily, Shyamli et al. (2021) generated a *de novo* long-read assembly for the cultivar Bhagya, predicting 32,062 protein-coding genes and, through integrated transcriptome profiling, identifying multiple heat-shock transcription factors and other candidate loci associated with drought stress responses.

Transcriptomic studies have further refined tissue- and species-level functional insights. Pasha et al. (2020) constructed cDNA libraries from leaf, root, stem, seed, and flower tissues of var. Bhagya, predicting 17,148 gene models and documenting high expression of enzymes involved in quercetin and kaempferol biosynthesis, vitamin pathways in leaves, flowers, and seeds, and metal-transport and storage proteins in roots. More recently, Shafi et al. (2022) produced comparative transcriptome profiles of *M. oleifera* and *M. concanensis*, revealing differential expression patterns that correlate with their reported anti-hyperglycemic activities.

The omics studies in moringa are still at an early stage but hold strong future potential. Current genomic, transcriptomic, and metabolomic data provide valuable insights into genes associated with yield, nutrition, stress tolerance, and bioactive compounds, thereby improving the understanding of the genetic and biochemical makeup of moringa. In the future, the integration of multiple omics approaches with advanced breeding and gene editing will enable more precise identification of useful genes and facilitate the development of improved varieties.

A further limitation is that moringa genomic, transcriptomic, and metabolomic datasets remain largely disconnected rather than integrated into a coherent systems framework. A systems-biology approach that integrates these layers via co-expression and metabolic network analyses would enable prioritization of candidate ANF genes based on their positions within regulatory and metabolic networks, thereby distinguishing pathway-specific tailoring enzymes from hub genes shared with primary metabolism. Such integration would move the current homology-based candidate lists in **Table 4** toward network-informed, testable target sets and represent an important emerging direction for moringa improvement.

**Table 4:** Putative ANFs biosynthesis candidate genes in *M. oleifera*.

S. No.	Anti-nutritional compounds	Biosynthesis pathway	Enzyme	Moringa transcript id	Homology (% identity/coverage)
1	Tannins	Flavonoid pathway	phenylalanine ammonia lyase	*Morol04g06840^b^	87.93% id; 100% cov to *CpPAL1*
			cinnamate 4-hydroxylase	***MoBGI036277g0264.1^a^	80.95% id; 99% cov to *AtC3H*
				***MoBGI036472g0011.1^c^	86.51% id; 100% cov to *BnC4H*
			4-coumarate-CoA ligase	*Morol01g09670^b^	73.32% id; 100% cov to *Cp4CL*
			chalcone synthase	*MoBGI036435g0009.1^c^	85.13% id; 98% cov to *BnCHS3*
			chalcone isomerase	*Morol13g04870^a^	67.74% id; 91% cov to *AtCHI*
			flavonoid 3-hydrolase	*MoBGI016008g0001.1^a^	80.49% id; 100% cov to *AtF3H*
			dihydroflavonol reductase	***MoBGI036102g0258.1^a^	72.31% id; 93% cov to *AtDFR*
			leucoanthocyanidin dioxygenase	*Morol11g04170^e^	100% id; 100% cov to *MoLDOX*
			leucoanthocyanidin reductase	*Morol01g03950^d^	69.58% id; 85% cov to *VvLAR*
			anthocyanidin reductase	*Morol12g02930^d^	78.18% id; 98% cov to *VvANR*
2	Saponins	Mevalonate pathway	farnesyl pyrophosphate synthase	*Morol01g32000^a^	81.52% id; 100% cov to *AlFPS*
			beta amyrin synthase	*Morol10g07740^a^	79.31% id; 99% cov to *AtBAS*
			squalene epoxidase 2/3	*Morol05g15470^a^	79.88% id; 93% cov to *AtSQE*
				*Morol02g05840^a^	77.59% id; 85% cov to *AtSQE*
			squalene synthase 1	*Morol02g15400^a^	78.35% id; 86% cov to *AtSQS1*
3	Glucosinolates	Glucosinolate pathway	branched-chain aminotransferase 3	*Morol02g05750^a^	76.68% id; 100% cov to *AtBCAT3*
			branched-chain aminotransferase 4/ branched-chain aminotransferase 6	**MoBGI036143g0043.1^a^	71.88% id; 99% cov to *AtBCAT4/ AtBCAT6*
			isopropyl malate isomerase large subunit 1	*Morol08g05500^a^	84.09% id; 100% cov to *AtIIL1*
			isopropyl malate dehydrogenase	*Morol01g30980^c^	83.50% id; 100% cov to *BnIMDH*
				*Morol11g05590^b^	73.33% id; 81% cov to *CpIMDH*
			methylthioalkylmalate synthase	*Morol01g33750^a^	81.41% id; 91% cov to *AtMAM*
			cytochrome P450 83B1	*Morol05g01530^b^	77.45% id; 96% cov to *CpCYP83B1*
				*Morol05g01540^b^	80.08% id; 100% cov to *CpCYP83B1*
			glutathione S-transferase U19	***MoBGI036109g0057.1^a^	75.93% id; 98% cov to *AtGSTU19*
			S- alkyl-thiohydroximate lyase	*Morol08g17210^b^	64.58% id; 97% cov to *CpSUR1*
			UDP-glycosyltransferase 74B1	**MoBGI036338g0268.1^b^	70.56% id; 94% cov to *CpUDP74B1*
			cytosolic sulfotransferase	***MoBGI036273g0112.1^a^	70.93% id; 96% cov to *AtSOT*
			flavin monooxygenase	*Morol11g05000^b^	78.23% id; 91% cov to *CpFMOGS- OX*
			UDP- rhamnose:rhamnosyltransferase 1	*Morol02g04890^d^	60.56% id; 90% cov to *VvRhaT1*
			5’-adenylsulfate reductases	*Morol03g21050^b^	72.48% id; 99% cov to *CpAPR5*
				*Morol05g13450^b^	76.85% id; 100% cov to *CpAPR4*
				*Morol08g02120^b^	71.06% id; 89% cov to *CpAPR1*
				*Morol10g14100^b^	78.49% id; 97% cov to *CpAPR3*
				*Morol12g01880^b^	87.1% id; 95% cov to *CpAPR1*
			ATP-sulfurylase	*Morol08g07340^b^	87.18% id; 100% cov to *CpAPS1*
				*Morol02g00230^a^	77.51% id; 87% cov to *AtAPS1*
			glutamate-cysteine ligase 1/ glutamate-cysteine ligase 2	*Morol11g03330^a^	80.34% id; 100% cov to *AtGSH1/2*
			O-acetylserine(thiol)lyase 1	*Morol03g18660^a^	81.07% id; 99% cov to *AtOASA1*
4	Phytic acid	Myoinositol pathway	inositol 1,3,4-trisphosphate 5/6- kinase	*Morol09g00240^d^	60.63% id; 100% cov to *VvITPK4*
			inositol 1,3,4-trisphosphate 5/6- kinase 3	***MoBGI036103g0067.1^a^	80.29% id; 96% cov to *AtITPK3*
			inositol polyphosphate kinase 2 beta	*Morol01g11380^a^	61.69% id; 94% cov to *AtIPK2beta*
			ATP-binding cassette C5	***MoBGI000954g0462.1^a^	86.75% id; 100% cov to *AtMRP5*
			phospholipase d a 1	*Morol10g03140^b^	88.24% id; 100% cov to *CpPLDALPHA1*
			regulator of chromosome condensation (RCC1) family with FYVE zinc finger domain	*Morol03g12400^d^	89.72% id; 100% cov to *VvPRAF1*
			myo-inositol-1-phosphate synthase	***MoBGI036201g0148.1^c^	92.55% id; 100% cov to *BnMIPS*

Confidence level is based on pairwise sequence identity and alignment coverage relative to the closest characterized ^a^*Arabidopsis thaliana*, *^b^Carica papaya, ^c^Brassica napus* , *^d^Vitis vinifera* and ^e^*Moringa oleifera* protein. Protiens were identified by BLAST homology to *Arabidopsis thaliana* and other model species; these are putative assignments may require experimental validation in moringa (e.g. expression profiling, reverse genetics, enzyme assays).

*Indicates genes with positive gene expression in 5 moringa tissues which includes flower, leaf, root, seed, stem (Chang et al., 2022; Pasha et al., 2020). Dataset available at https://bioinformatics.psb.ugent.be/gdb/aocc/morolbgi/Moringa_version2/.

**Indicates genes with positive gene expression in 4 moringa tissues, including flower, leaf, seed, and stem (Guo et al., 2026).

***Indicates genes that still require functional validation. Dataset available at https://bioinformatics.psb.ugent.be/orcae/aocc/overview/MorolBGI

## ANFs and their reduction strategies

### ANFs in moringa and their health impacts

Moringa is highly valued for its nutritional quality because its leaves, seeds, and flowers provide proteins, fiber, vitamins A, B, C, and E, and minerals such as iron, calcium, potassium, magnesium, zinc, and phosphorus. Moringa contains a favorable amino acid profile, including sulfur-containing amino acids, which makes it especially useful as a plant-based supplement in balanced diets. The favorable functional characteristics of proteins from moringa (*viz*, solubility, emulsification, foaming) make it a better candidate for processing industries for biofortification. Biofortifying moringa leaf powder can improve the nutrient density of cereal-based foods, breads, snacks, pasta, beverages, and dairy or meat products, helping address malnutrition and undernutrition. However, consumer acceptance of moringa-fortified foods is often limited by undesirable sensory traits, such as the addition of moringa powder or extracts, which impart a green color, flavor, and texture to the products. In particular, moringa can impart a strong green color and bitterness, which lowers overall acceptability.

**Figure 4:**
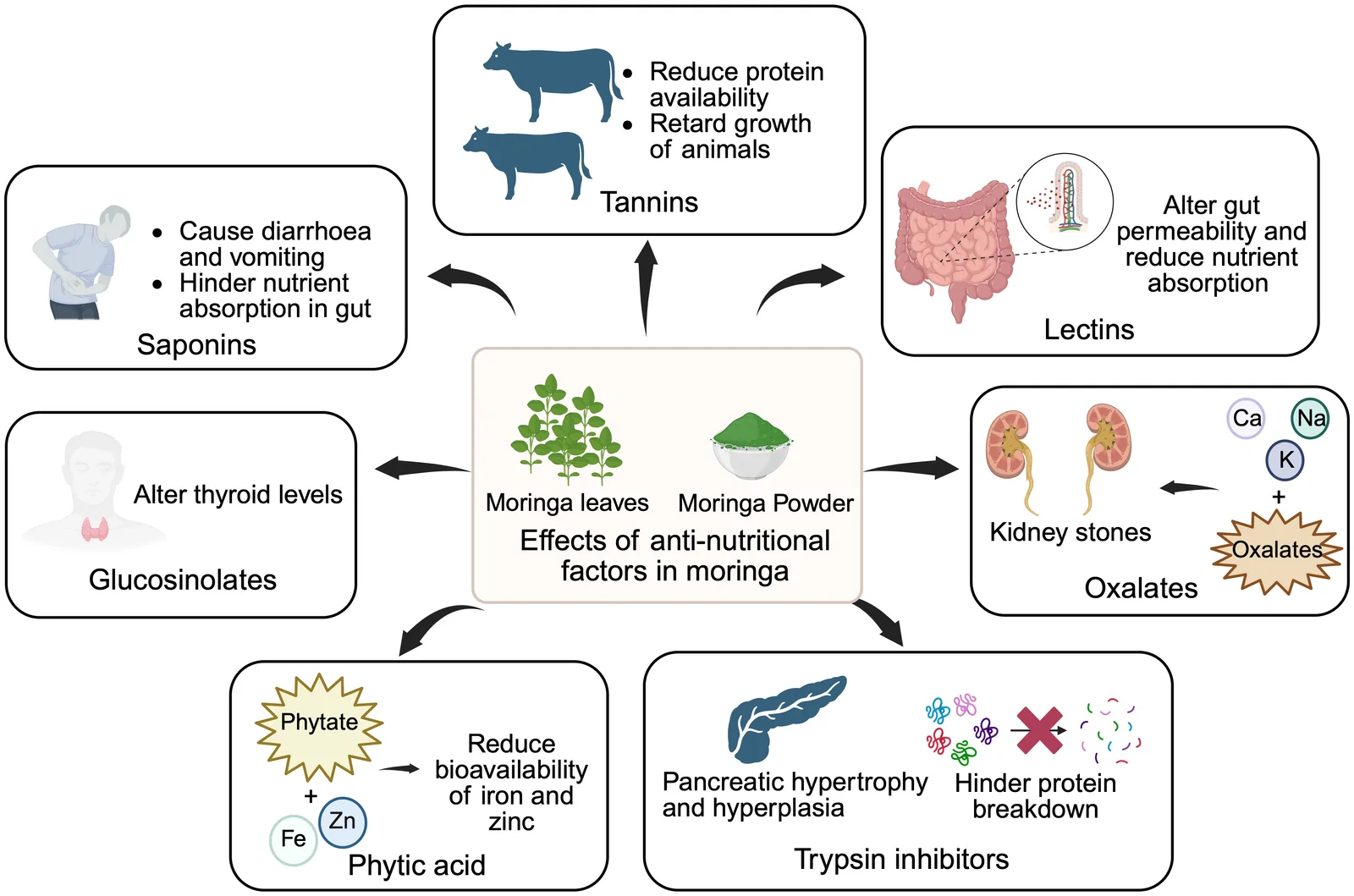
Effects of anti-nutritional factors (ANFs) in *M. oleifera*. Created in BioRender. Kanagarajan, S. (2026) https://BioRender.com/ic2pj2o.

These off-flavors are closely linked to the presence of ANFs, including alkaloids, tannins, saponins, GSLs, lectins, oxalates, phytates, and protease inhibitors, which, at high inclusion levels, may also reduce mineral bioavailability, impair protein digestibility, and palatability which contradicts the initial purpose on nutritional aspects (**Figure 4**) (Leone et al., 2015; Makkar and Becker, 1996, 1997; Thurber and Fahey, 2009). **Figure 4** summarizes these effects, showing that moringa’s ANFs act through several distinct mechanisms, from mineral chelation and protein complexing to enzyme inhibition and thyroid interference, which is why no single processing step neutralizes all of them and why a combination of strategies is required. This section summarizes the effects of these major ANFs in moringa on nutrient bioavailability, digestion, and overall nutritional value.

The ANFs differ markedly in the severity of their effects, and several are not uniformly detrimental. GSLs and their hydrolysis products are among the most consequential because of their potential to impair thyroid function (Tripathi and Mishra, 2007), whereas oxalates are primarily of concern in susceptible individuals, particularly when high intake coincides with low dietary calcium and increases the risk of calcium oxalate kidney stone formation (Crivelli et al., 2020). Tannins and phytates have more moderate, dose-dependent effects on protein and mineral bioavailability; however, both also possess beneficial properties, including anti-inflammatory, antibacterial, and anticancer activity for tannins and antioxidant activity for phytate. Accordingly, the objective should be to reduce these compounds to safe, nutritionally acceptable thresholds rather than eliminate them completely. Lectins and trypsin inhibitors are of greatest concern in seed-derived products and can be substantially reduced by thermal processing. Collectively, this gradient of severity, together with the dual nutritional roles of several compounds, supports a strategy focused on rebalancing ANF composition rather than abolishing ANF content.

#### Alkaloids

Moringa contains several alkaloids with both beneficial and potentially adverse effects. Despite toxicity risks (upon excessive consumption), moringa alkaloids such as N-α-L-rhamnopyranosyl vincosamide and deoxy-niazimicin offer benefits like cardiovascular protection and free radical scavenging from compounds. Also, root extracts show antitumor activity against lung cancer cells in lab studies. However, excessive intake of bark alkaloids, including moringinine and moringine, has been reported to alter clotting factors, modify serum composition, and inhibit blood transferase activity in animal studies at doses of 500 mg/kg and 184 mg/kg, respectively, while some alkaloids can cause neuromuscular effects such as nerve paralysis (Abd El-Hack et al., 2018). The characterization of alkaloid biosynthetic pathways in moringa, along with identification of key regulatory genes and the application of omics technologies and metabolic engineering, could enable precise regulation or enhancement of beneficial alkaloid production while minimizing associated toxicity. Therefore, the targeted studies on the optimal level of moringa consumption are warranted.

#### Tannins

Tannins in moringa show a dual role, acting as both beneficial bioactive compounds and ANFs depending on their concentration. Tannins (C_76_H_52_O_46_) are water-soluble phenolic compounds reported in moringa. Dried leaves have a maximum of 13.2 g of tannins per kg of leaf (Bhatta et al., 2012), while the content is low in the freeze-dried leaves (5–12 g/kg) (Makkar and Becker, 1997; Richter et al., 2003). The appreciable amounts of tannins in moringa possess medicinal properties like anti-cancer, anti-atherosclerotic, anti-inflammatory, anti-hepatotoxic, and antibacterial. Also, it inhibits HIV replication activity (Kancheva and Kasaikina, 2013). A higher concentration of tannins reduces feed intake capacity, has a reduced growth rate, and reduces protein digestibility (Chung et al., 1998; Makkar, 2003). In cattle, it reduces feed intake, palatability, and digestibility (Akhtar et al., 2022). It binds and precipitates the proteins and alkaloids. Metabolic engineering and precision breeding may enable the development of improved genotypes with balanced tannin levels. Further, pharmacological studies and controlled extraction methods could enhance their safe use in nutraceutical and pharmaceutical applications.

#### Saponins

The saponin (C_58_H_94_O_27_) in moringa gives a bitter taste to livestock (Makkar and Becker, 1996, 1997). Saponin is categorized as a steroidal and triterpenoid type, and both are produced through the mevalonate pathway. Triterpenoid saponins are seen predominantly in the dicots, while steroidal saponins mostly prevail in the monocots (Moses et al., 2014). Saponins are glycosides in which one or more sugar moieties are covalently linked to an aglycone (sapogenin) (Augustin et al., 2011). A considerable amount of saponin exhibits anticancer properties, and some of it has hemolytic side effects (Man et al., 2010; Tian et al., 2013). The concentration of saponin in dried leaves of moringa was recorded as 50 g/kg (Makkar and Becker, 1996), whereas in freeze-dried leaves it ranged between 64 g/kg and 81 g/kg (Makkar and Becker, 1997; Richter et al., 2003). It has both positive (Immunostimulant, hypocholesterolemic, antifungal, antiviral, and anticarcinogenic) and negative effects (diarrhea, vomiting, and hindered nutrient absorption in the gut) if consumed more than the recommended level in animals (Duraiswamy et al., 2023). Future research should focus on detailed characterization of saponin biosynthetic pathways, regulation of key genes in the mevalonate pathway, and identification of structural variants responsible for beneficial and toxic effects. In addition, improved extraction techniques, formulation strategies, and dose standardization will be essential to fully exploit their pharmacological potential in safe and effective applications.

#### Glucosinolates

GSLs are sulfur- and nitrogen-containing compounds synthesized from glucose and amino acid precursors, consisting of a thio-glucose and a sulfonated oxime linked to an extended amino acid chain (Blazevic et al., 2020). They are frequently observed in Brassicaceae members, including rapeseed, cabbage, and mustard. GSLs undergo hydrolysis by myrosinases, producing isothiocyanates and epithionitriles (Wittstock et al., 2003). The common GSLs in moringa are glucomoringin (4-[α-L-rhamnosyloxy)-benzyl]-GSL, which is found in all the tissues, and glucotropaeolin (Benzyl GSL), specifically found in roots. Apart from these compounds, Sinalbin (4-hydroxy benzyl), 4-O-(α-L-acetylrhamnopyranosyloxy)-benzyl isomer 1, 4-O-(α-L-acetylrhamnopyranosyloxy)-benzyl isomer 2, 4-O-(α-L acetylrhamnopyranosyloxy)-benzyl isomer three are also found in various tissues in moringa (Amaglo et al., 2010). GSLs have been reported to disrupt thyroid hormone levels in humans (Tripathi and Mishra, 2007). The pungency of GSLs decreases consumer preference and palatability of fodder.

#### Lectins

Lectins in moringa are primarily found in seed extracts or kernels and possess carbohydrate-binding sites to bind with sugar (Makkar and Becker, 1997). It is a major ANF due to its ability to alter gut permeability and reduce the efficient absorption of nutrients by the gut epithelium (Alatorre-Cruz et al., 2018). Moreover, it can coagulate red and white blood cells, necessitating strict monitoring of consumption levels (Araujo et al., 2013).

#### Oxalates and phytates

The oxalate and phytate concentrations in the dried leaves of moringa range from 334.33 mg/100 g (Joshi and Mehta, 2010) and 25-31 g/kg (Teixeira et al., 2014), respectively. Oxalates bind to water-soluble salts, including calcium, sodium, and potassium, thereby reducing their bioavailability. More oxalic acid in a diet with limited calcium leads to the formation of kidney stones (Crivelli et al., 2020).

Phytic acid (C_6_H_18_O_24_P_6_) is a major ANF exhibiting chelate formations with positively charged ions like iron and zinc, thus reducing their bioavailability in the human system (Schlemmer et al., 2009). It is an important form of storage phosphates found in plants. Monogastric animals lack phytase to digest phytic acid, thus leading to reduced absorption of micronutrients. Therefore, it is essential to reduce the phytate content to improve the mineral availability in the system. Moringa leaves have 25 g/kg of phytate in dried leaves (Makkar and Becker, 1996).

#### Trypsin inhibitors

Trypsin is the major class of enzymes involved in protein catabolism. Trypsin inhibitors modify the trypsin activity in the gut and interfere with protein breakdown. They bind to trypsin and inhibit protein digestion, thereby affecting amino acid absorption. The leaf flour contains 1.45 mg/100 g of trypsin inhibitor (Makkar and Becker, 1997). The trypsin inhibitor from *M. oleifera* flowers (MoFTI) exhibits anti-inflammatory effects (Patriota et al., 2021). Regular feeding of purified trypsin inhibitors induces pancreatic hypertrophy and hyperplasia in monogastric animals. Although these compounds may have beneficial biological activities at low levels, their anti-nutritional effects highlight the need for proper processing and breeding strategies to improve the nutritional quality of moringa.

### Traditional processing methods and their limitations

Traditional chemical and physical methods, such as heating, cooking, grinding, milling, soaking, and germination, are commonly used to reduce ANFs in plant-based foods; however, these techniques are often inefficient and can compromise nutrient quality (Samtiya et al., 2020). The elimination of ANFs is essential for safe human consumption. The traditional methods used to reduce ANFs are presented in **Table 5**; heating, boiling, and soaking are the most commonly employed and the most economically accessible. As the comparison makes clear, however, these simplest methods are also the most prone to leaching water-soluble vitamins and minerals, so the practical choice of method involves a trade-off between ANF removal and nutrient retention rather than a single optimal technique. Typically, processing techniques such as drying, wet processing, chemical treatments and irradiation are applied to reduce ANFs; however, in moringa, these methods frequently decrease vitamin and mineral content (Mehlomakulu and Emmambux, 2022; Razzak et al., 2022). Soaking or extraction leads to substantial losses of water-soluble vitamins (e.g., vitamin C) and minerals (e.g., calcium and potassium) (Mehlomakulu and Emmambux, 2022; Gidamis et al., 2003). Drying and powdering leaves, the most common preparation method for supplements, also fail to remove ANFs; the persistent presence of ANFs after various drying processes raises concerns about nutrient bioavailability (Ademiluyi et al., 2018). Thus, there is a persistent trade-off between reducing ANFs and preserving nutrients.

**Table 5:** Traditional methods of ANFs reduction in *M. oleifera*.

Method	Speed	Cost	Effect on nutrients	Overall implication	References
Heating (boiling, blanching)	Fast (minutes)	Low	Significant loss of heat- and water-soluble vitamins (vitamin C, B-complex); possible mineral leaching	Boiling reduced 85.3% oxalates, 85.4% phytates, and 78.8% trypsin inhibitors; while blanching reduced 20.7% oxalate and 39.8% phytate	Saini et al., 2016; Razzak et al., 2022; Sallau et al., 2012
Soaking (water)	Moderate (hours)	Very low	Leaching of minerals and water-soluble vitamins; minimal effect on heat-stable nutrients	Lowers phytate and tannin content of moringa seed flour; accompanied by leaching of soluble nutrients	Mehlomakulu and Emmambux, 2022; Saa et al., 2022; Matthew et al., 2022
Germination (sprouting)	Slow (days)	Very low	Generally nutrient- preserving or nutrient- enhancing; can raise protein and mineral availability	Reduces phytate, tannins, and saponins in moringa seed flour while improving functional and nutritional properties	Saa et al., 2022; Ijarotimi et al., 2013
Drying and milling (leaf powder)	Moderate (hours)	Low–moderate	Alters phenolic and antioxidant content; some micronutrient loss depending on drying method	Drying does not fully remove ANFs. ANFs persist in moringa leaf powder after common drying methods	Ademiluyi et al., 2018; Mehlomakulu and Emmambux, 2022
Microbial fermentation	Moderate (hours–days)	Low–moderate	Typically increases protein, amino acids, phenolics and mineral bioavailability	50-70% reduction of phytate, 40-57% reduction of tannins, and 23% reduction of glucosinolates in moringa	Shi et al., 2020, 2021, 2022; Thierry et al., 2013
Alkali treatment	Moderate	Low	Possible leaching of soluble proteins	Reduced major anti-nutrients including phytic acid, glucosinolates, trypsin inhibitors	Devisetti et al., 2016
Irradiation	Fast	Moderate–high	Variable; can preserve or degrade nutrients depending on dose and method	Not yet quantified in moringa; reported for other crops but moringa-specific data are lacking	Maetens et al., 2018; Kadam et al., 2021

Engineering plants with lower intrinsic ANF levels or applying microbial fermentation to degrade ANFs can mitigate this trade-off by reducing the need for harsh processing while maintaining nutrient density. The following sections critically evaluate microbial fermentation and genome-editing-based strategies for reducing ANFs in moringa.

### ANFs reduction strategies: microbial fermentation

Fermentation, a biological process using bacteria, fungi, or yeast, transforms complex organic compounds into simpler forms and can reduce ANFs via enzymatic degradation, acidification, and microbial metabolism, while improving nutritional and sensory attributes (Demir et al., 2006; Lucke et al., 2019). While microbial fermentation is currently the most practical and scalable near-term strategy for reducing ANFs in moringa products, genome editing represents a complementary long-term solution that can lower ANF levels directly in the plant, thereby reducing reliance on intensive processing (Shi et al., 2021).

In moringa leaves and seeds, solid-state fermentation with fungi and mixed microbial consortia consistently reduces the major ANFs tannins, phytic acid, and GSLs while increasing protein content and antioxidant activity. For example, *Aspergillus niger*, *Candida utilis*, and *Bacillus subtilis* fermentation of moringa leaf meal decreased tannins and phytic acid markedly and lowered GSL levels, while enhancing crude protein and phenolics (Shi et al., 2021). In moringa seeds, fermentation reduced phytates, tannins, alkaloids, and saponins (Ijarotimi et al., 2013). Lactic acid fermentation of moringa leaf powders has achieved 60–67% reductions in phytate content and substantial improvements in iron availability, indicating that fermentation is particularly effective against hydrolyzable ANFs such as phytate and tannins (Thierry et al., 2013).

The microbial fermentation process could improve the overall nutritional profile by increasing the bioavailability of vitamins and minerals and enhancing the digestibility of food products (Shahidi and Zhong, 2010). Fermentation also enriches amino acid content, especially lysine, tryptophan, and methionine, making it a sustainable way to biofortify amino acids and other essential nutrients (Mohapatra et al., 2019). Additionally, it promotes antimicrobial, antioxidant, and anticancer activities in humans by increasing the levels of phenolic compounds and the free-radical scavenging potential (Ali et al., 2020).

Most plant-based food fermentation processes use lactic acid bacteria (*Lactobacillus* spp. and *Streptococcus* spp.), *Bacilli*, fungi (*Aspergillus* spp.), and yeast (*Saccharomyces cerevisiae*) to degrade ANFs, due to their ability to produce enzymes like protease, amylase, xylanase, and pectinase (Peng et al., 2025). Solid-state fermentation (SSF), which involves growing microorganisms on substrates with limited water, is commonly used in food fermentation and is also applied to moringa leaves with various organisms such as *Rhizopus oligosporus*, *A. niger*, *Bacillus* spp., and *C. utilis* to improve bioactive compounds and nutrition (Ijarotimi et al., 2013; Zhang et al., 2017b; Djonu et al., 2018; Dai et al., 2020; Feitosa et al., 2020; Shi et al., 2022). Nonetheless, research on reducing ANFs in moringa through fermentation remains limited, with few studies exploring this aspect. Using SSF with these microbes, whether as monocultures, consortia, or combinations, can notably decrease levels of ANFs such as phytic acid, tannins, and GSLs (Shi et al., 2022). However, the selection of microbial inoculum and the fermentation duration influence the quality of the final product (Mannaa et al., 2021).

Fermentation with lactic acid bacteria is the most common method used in the food industry for biofortifying foods with vitamins, minerals, and amino acids. In lactic acid bacteria fermentation, *Lactobacillus plantarum*, *L. fermentum*, and *L. sanfranciscensis* produce phytase enzymes, which reduce phytate levels and increase mineral bioavailability (Thierry et al., 2013). Fermentation with lactic acid bacteria in moringa-based beverages has been shown to lower raffinose content (Vanajakshi et al., 2015). Among these bacteria, *L. plantarum*, *L. paraplantarum*, and *L. pentosus* can degrade tannins via the tannase enzyme, which cleaves tannins into glucose and gallic acid, thereby enhancing iron absorption (Rodriguez et al., 2009). Besides lactic acid bacteria fermentation, a well-known probiotic strain, *Bacillus subtilis*, increases the levels of total isoflavones, amino acids, and phenolics in moringa leaves (Ali et al., 2020). In addition to bacterial fermentation, industrially relevant filamentous fungi such as *Aspergillus* are major sources of tannase, phytase, and other enzymes that break down phytate, tannins, and GSLs, thereby improving nutrient bioavailability (Feitosa et al., 2020; Shi et al., 2022). Along with ANFs degradation, it enhances mineral bioavailability during yeast fermentation; several yeast strains, such as *S. cerevisiae*, *C. utilis*, *C. tropicalis*, and *Pichia kluyveri*, enhance calcium bioavailability (Dai et al., 2020). Recently, a study showed that fermentation with *Lactiplantibacillus* sp. and *S. cerevisiae* increased dietary folate levels in moringa (Du et al., 2023). In another study, SSF using fungi (*Rhizopus*, *Aspergillus*, and *Neurospora*) increased phenolic content and antioxidant activity after 24 hours of fermentation. However, prolonged incubation led to a significant reduction in these activities (Starzynska-Janiszewska et al., 2022). In addition to monoculture fermentation, combining two or more strains with diverse protease enzymes significantly reduces the levels of multiple ANFs, including phytic acid, GSLs, and tannins, while increasing antioxidants, phenolics, flavonoids, and protein quality in moringa leaf meal (Shi et al., 2020, 2022).

#### Challenges in fermentation application

Overall, fermentation is most effective for ANFs that are degradable by enzymes, such as phytate, tannins, and certain GSLs, often achieving 50–70% reductions while simultaneously improving mineral bioavailability and sensory quality (Samtiya et al., 2020; Sharma et al., 2020; Arsov et al., 2024; Murniece et al., 2025; Tomar et al., 2025). However, residual levels of some GSLs and oligosaccharides may remain, and the processing outcomes depend strongly on strain selection and fermentation duration; therefore, fermentation alone cannot fully eliminate all ANFs in moringa products. Taken together, these studies suggest a clear division of labor among fermentation approaches by ANF class, rather than a uniform benefit across all ANFs. Enzymatically degradable compounds, including phytate, tannins, and certain GSLs, are often reduced substantially by phytase-, tannase-, and protease-producing bacterial and fungal strains, whereas structurally protected or non-enzyme-substrate compounds generally show more limited reductions. Two broader patterns also emerge from the literature. First, fermentation outcomes appear to depend more on the enzyme repertoire of individual strains and fermentation duration than on the microbial species alone, with prolonged incubation in some cases diminishing the initial improvements (Starzyńska-Janiszewska et al., 2022). Second, multi-strain microbial consortia have frequently achieved greater simultaneous reductions across multiple ANFs than single-strain fermentations (Shi et al., 2020, 2022). However, reproducibility and scale-up remain important challenges. Most moringa-specific studies have been conducted at laboratory scale using diverse substrates, processing methods, and fermentation conditions, limiting direct comparison across studies. Consequently, there remains a need for systematic strain–substrate–time optimization and validation under standardized, food-grade processing conditions before these promising laboratory findings can be translated into robust commercial applications. This makes microbial fermentation the most feasible near-term strategy for reducing ANFs in moringa, while genome editing provides a complementary long-term option to directly lower specific ANF pathways in the plant, thereby decreasing reliance on intensive processing.

Beyond efficacy, fermentation carries practical drawbacks: outcomes are inconsistent because they depend on conditions, microbe selection, and duration (Zentek and Boroojeni, 2020); uncontrolled processes risk contamination and spoilage (Capozzi et al., 2017); prolonged fermentation can generate unwanted compounds and cause nutrient loss (Pessione, 2012; Grujovic et al., 2022); and the sensory changes it introduces, together with the infrastructure needed for controlled, food-grade production, may limit acceptance and raise cost (Marco et al., 2017; Tamang et al., 2020). Even so, the successful use of these strains across various crops underscores fermentation’s potential to improve moringa’s sensory quality, nutritional value, and biological properties.

### ANFs reduction strategies: genome editing

Genome editing has significant potential to ensure food and nutritional security for the population. Genome editing technologies enable us to manipulate DNA sequences to get desired mutants with desired phenotypes (Dhugga, 2022). Recent advances in new genomic techniques (NGTs), particularly CRISPR/Cas-based genome editing, offer opportunities to reduce ANFs in moringa while preserving desirable agronomical traits. It should be stated at the outset that, to date, no CRISPR-based genome editing has been reported in moringa. The strategies discussed in this section are therefore extrapolated from results in other crop systems and are presented as emerging research directions rather than established or near-term applications in moringa. CRISPR (Clustered Regularly Interspaced Short Palindromic Repeats) uses a guide RNA sequence to target specific DNA sequences and the endonuclease Cas9 to cut DNA at the target site (Cong et al., 2013), which makes site-specific manipulation of DNA possible. Other genome-editing tools, such as TALENs (Transcription Activator-Like Effector Nucleases) and zinc finger nucleases, are surpassed by CRISPR due to its greater precision and specificity in modifying gene sequences (Gonzalez Castro et al., 2021).

With the growing availability of moringa genomic resources strengthens the use of CRISPR-based crop improvement. Although gene annotation in moringa is still incomplete, chromosome-scale genome assemblies and emerging functional data now provide a foundation for identifying candidate genes linked to ANFs and quality traits. Consequently, genome editing may serve as a complement to conventional breeding by enabling targeted reduction of undesirable compounds while preserving or enhancing nutritional and pharmacological properties in improved moringa cultivars.

#### Advances in genome editing

*Agrobacterium tumefaciens* is a gram-negative bacterium and a widely deployed tool for genome editing. The presence of the Ti plasmid makes it a biological tool to transfer the genetic fragments. Transfection is done by replacing the T-DNA region with the gene of interest and co-cultivating the bacteria with the explant. CRISPR, along with *Agrobacterium*, has revolutionized genome editing, in which the former enables precise DNA editing in the host, and the latter delivers CRISPR-related genes. A successful *Agrobacterium* transformation was achieved in moringa by Zhang et al. (2017a). Notably, this study demonstrated gene delivery rather than CRISPR editing, and no regeneration of edited moringa plants has yet been reported; it therefore establishes the feasibility of transformation but not of editing in this species. However, the integration of bacterial DNA into the plant genome affects public acceptance. Additionally, eliminating transgene segments is nearly impossible in perennial and vegetatively propagated plants. Transgene-free genome editing could be the best alternative for this approach. Ribonucleoprotein (RNP) delivery, in which the Cas9 enzyme is pre-complexed with guide RNA, overcomes plasmid integration and consumer acceptance concerns: it reduces off-target effects and random DNA integration, requires no vector construction, and faces lighter regulatory scrutiny because no foreign DNA is integrated (Woo et al., 2015). However, this requires developing an efficient protoplast regeneration protocol, which is currently lacking in most crops, including moringa.

#### Genome editing through grafting

Moringa has demonstrated promising regenerative capacity in tissue culture, and micropropagation has been widely used for clonal multiplication and for rescuing endangered species (Saini et al., 2012; Steinitz et al., 2009; Stephenson and Fahey, 2004; Zheng et al., 2022). However, *in vitro* conditions may induce stress in explants, thereby inducing antioxidant enzyme expression and negatively affecting regeneration (Hassanein et al., 2018; Boopathi et al., 2021). In addition, responses to *in vitro* propagation are highly genotype- and explant-dependent. Moreover, different ecotypes and explants require different tailored media and supplements for successful regeneration (Stephenson and Fahey, 2004; Saini et al., 2012; Gupta et al., 2020). Even under optimized protocols, moringa cultures are prone to hyperhydricity, leading to vitrification and shoot malformation, which further complicates the development of efficient regeneration systems for genome-editing applications (Boopathi et al., 2021). Thus, optimizing protocols for successful moringa regeneration is the first step toward successful genome editing.

**Figure 5:**
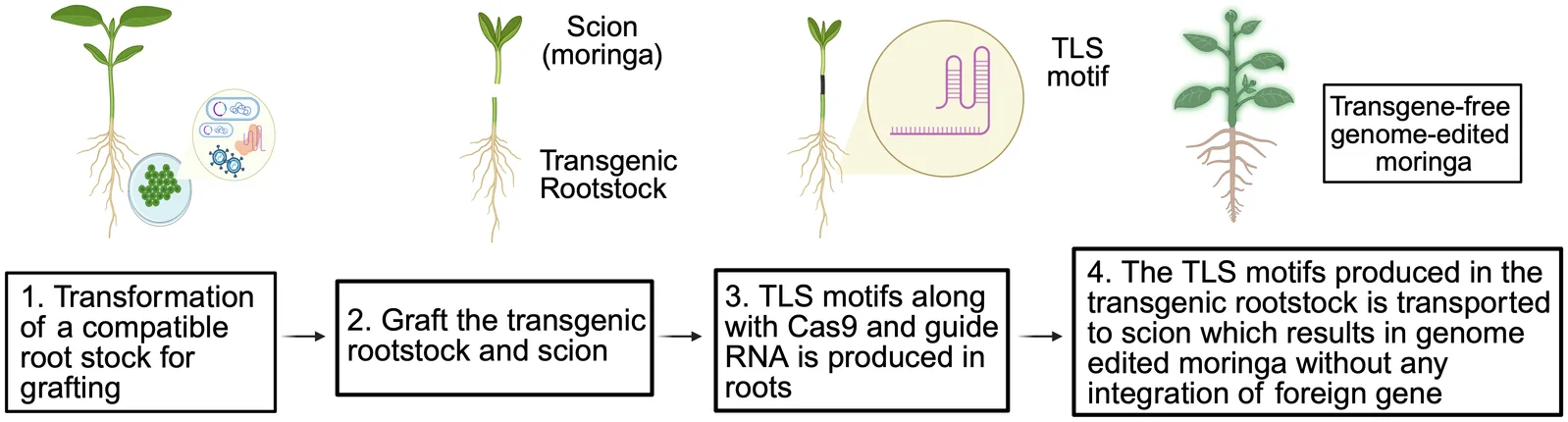
Transgene-free genome editing in *M. oleifera* via grafting: a transformed rootstock expresses mobile Cas9-gRNA-TLS complexes that move through the graft union to edit the wild-type scion, yielding transgene-free edited seed. Created in BioRender. Kanagarajan, S. (2026) https://BioRender.com/ecrhcwy.

Recent advances in genome editing through grafting could be a promising alternative for moringa. The schematic representation of the genome editing approach through the grafting technique is presented in **Figure 5**. In this, Yang et al. (2023) described a strategy to obtain transgene-free edited plants by using a genetically engineered rootstock expressing Cas9 and guide RNAs fused to transfer-RNA-like sequence (TLS) motifs, which are capable of long-distance movement through the vascular system in model species. Whether this approach transfers to a woody perennial such as moringa, including graft compatibility and edit efficiency across the graft union, remains to be tested.

In this approach, a compatible rootstock is first transformed via RNP delivery, *Agrobacterium*, or viral vectors to express a Cas9–gRNA–TLS complex. Then the transformed rootstock and scion (moringa) are grafted. This graft union would, in principle, facilitate long-distance transport of TLS motifs into the moringa scion, where they could introduce targeted edits. If successful, this would yield transgene-free genome-edited moringa, with seeds from the edited scion inheriting the desired mutations while remaining free of foreign genes.

This technique eliminates the pressure of *in vitro* culture and protocol optimization, as any compatible rootstock responsive to tissue culture can be utilized to develop a rootstock. The compatible rootstock and the scion could then be grafted to achieve the desired genome editing in the scion. Similarly, it eases the regulatory and consumer concerns associated with transgene integration and could accelerate the development of genome-edited, low-ANF, high-nutrient cultivars. However, the technique requires the transformation of rootstocks for each gene of interest and does not apply to crops for which a grafting protocol has not been established (Hu and Gao, 2023). Moreover, efficient multiplex editing of multiple traits via a single engineered rootstock remains largely unexplored and warrants further investigation.

### Tailoring plant secondary metabolism through promoter editing

ANFs are secondary metabolites that play a major role in plant defense against biotic and abiotic stresses. Secondary metabolites are compounds derived from primary metabolites to support additional functions, mainly protection against herbivores, pests, and pathogens, which are essential for plant survival (Harborne, 1990). The aim of reducing ANFs through biotechnological strategies should focus on altering metabolite profiles. The complete knockout might affect the distribution of these compounds in the plants. This may compromise the plant’s existing defense system, leading to plant death or reduced quality and yield. Thus, reducing ANFs in moringa should be undertaken to preserve plant defense.

To target moringa ANFs, we suggest promoter editing as a means to alter gene expression rather than knock them out completely. The promoter responsible for gene regulation is located upstream from the region to be transcribed (Porto et al., 2014). It is the site where RNA polymerase binds to start transcription. Manipulating the promoter region directly affects gene expression. In this case, manipulating promoter regions might alter gene expression sufficiently to maintain the defense system while reducing it to meet consumer preferences. Promoter editing has been successfully implemented in staple crops such as rice (Cui et al., 2020; Huang et al., 2020) and maize (Liu et al., 2021) and has yielded the expected results. In these cases, editing *cis*-regulatory regions produced quantitative, fine-tuned changes in gene expression rather than the all-or-nothing loss of a knockout: promoter editing of the Wx gene generated a continuous range of amylose levels and improved grain quality in rice (Huang et al., 2020), CRISPR editing of a yield-related QTL promoter created beneficial allelic variation (Cui et al., 2020), and promoter editing of CLE genes produced graded improvements in grain-yield traits in maize (Liu et al., 2021). These examples demonstrate that promoter editing can dial gene expression up or down to a desired level, which is precisely the control needed to lower an ANF while retaining the residual expression required for plant defense. Its application to moringa is, however, hypothetical at this stage and is proposed here as a future direction. Promoter regions have previously been modified using CRISPR/Cas technology via *Agrobacterium*-mediated transformation. We propose promoter editing using RNPs to achieve a higher transformation rate and prevent the integration of foreign DNA, thereby easing public acceptance and addressing ethical concerns.

### Biosynthesis pathways of major ANFs and candidate genes for their reduction

Genome editing depends on existing genetic information. A major challenge in genome editing in moringa is the limited publicly available genomic data with incomplete annotations. The recently published moringa genome and transcriptome data (Chang et al., 2022) provide an essential framework for molecular breeding, but the genome assembly is still only partially annotated, and many gene models lack functional assignment. This limited annotation constrains immediate genome editing applications because candidate genes for ANFs often have to be inferred indirectly from homology rather than experimentally confirmed function. As a result, target selection currently relies heavily on orthology to *Arabidopsis* and other model species, increasing the risk of off-target physiological effects if edited genes participate in multiple pathways. The lack of comprehensive functional annotation makes it difficult to prioritize genes with narrow, pathway-specific roles in ANF biosynthesis versus genes that are also involved in primary metabolism, stress responses or developmental regulation.

The AOCC moringa genome and transcriptome are accessible via the ORCAE-AOCC portal hosted at VIB–UGent (https://bioinformatics.psb.ugent.be/gdb/aocc/morolbgi/Moringa_version2/). The comparative genomic study revealed a close phylogenetic relationship of moringa with *Arabidopsis thaliana*, papaya (*Carica papaya*), barrel clover (*Medicago truncatula*), grapevine (*Vitis vinifera*), tomato (*Solanum lycopersicum*), lotus (*Nelumbo nucifera*), rice (*Oryza sativa* L. subsp. *japonica*), maize (*Zea mays*), avocado (*Persea americana* Mill.), and *Amborella trichopoda* (Chang et al., 2022). A total of 349,936 protein sequences from the genomes of these 11 species were compared, revealing 5,185 orthogroups shared by all. Blasting the transcriptomic data with closely related species identified putative orthologs of genes associated with ANF biosynthesis in moringa (**Table 4**). These candidate orthologs could represent starting points for genome-editing studies aimed at modifying ANF profiles. As **Table 4** indicates, these assignments rest on BLAST homology to Arabidopsis and other models rather than on function demonstrated in moringa, so the table is best read as a prioritized list of hypotheses for functional testing rather than a catalog of confirmed ANF genes. Across the genomic, transcriptomic, and metabolomic studies surveyed, moringa research has progressed rapidly from an initial draft reference to multiple independent genome assemblies, but the marked differences in predicted gene number and annotation frameworks indicate that these resources are not yet fully harmonized across studies (Chang et al., 2019, 2022; Shyamli et al., 2021; Tian et al., 2015). As a result, moringa omics currently supports candidate and hypothesis generation more strongly than confident editing-target selection, because many ANF-relevant genes still lack direct functional validation and are prioritized mainly through homology-based inference. The most valuable next step is therefore the integration of existing genomic, transcriptomic, and metabolomic resources into a unified, functionally annotated pan-genome framework in which candidate ANF genes are experimentally validated against phenotype.

**Figure 6:**
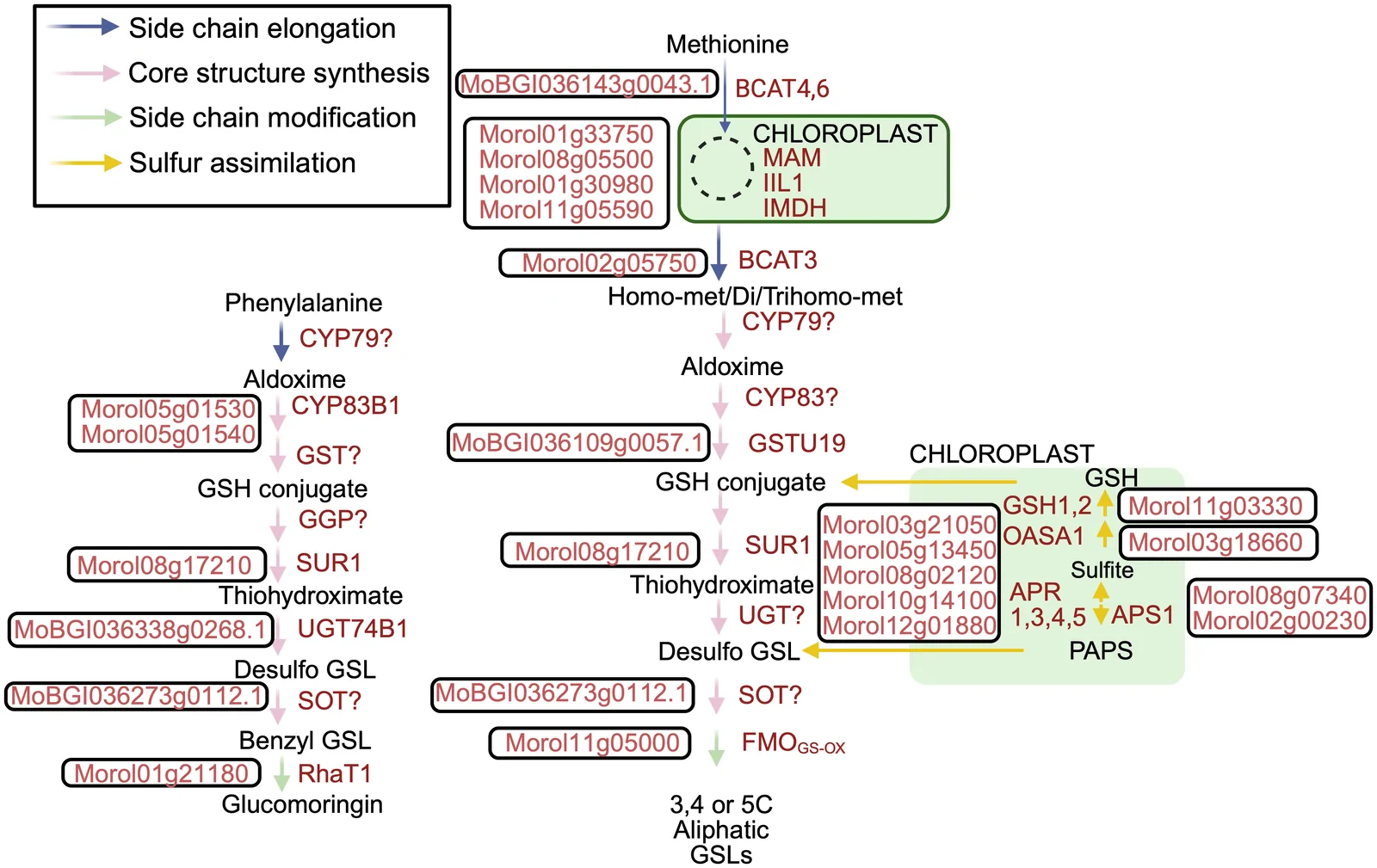
Predicted glucosinolate biosynthesis pathway of *M. oleifera*. Created in BioRender. Kanagarajan, S. (2026) https://BioRender.com/sodarb9.

**Figure 7:**
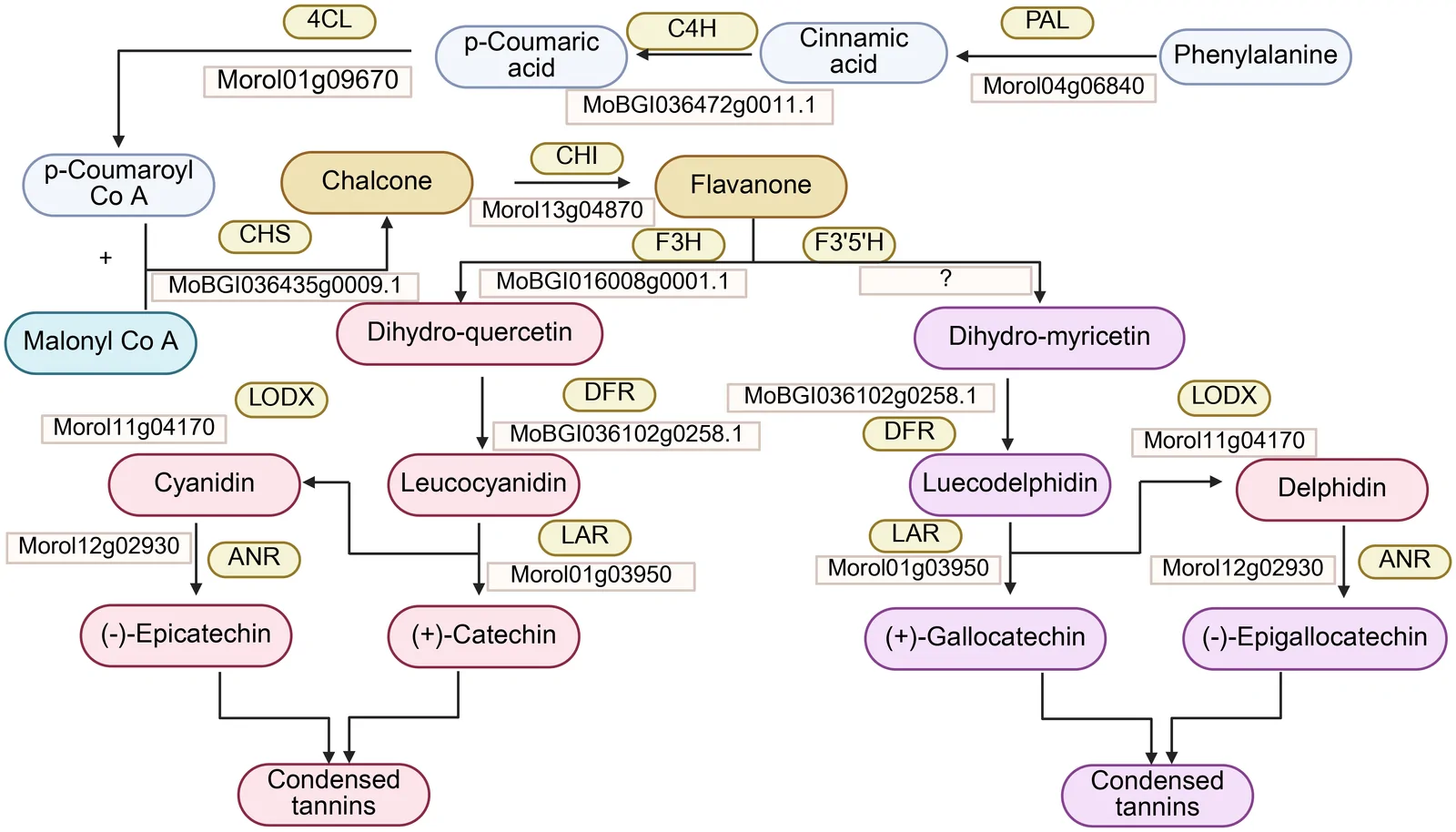
Predicted tannin biosynthesis pathway of *M. oleifera*. Created in BioRender. Kanagarajan, S. (2026) https://BioRender.com/iphct4j.

A predicted biosynthetic pathway of the major ANFs in moringa is schematically represented in **Figures 6**, **7**: tannins are synthesized through the phenylpropanoid pathway as polymerized flavonoid derivatives (**Figure 7**), whereas GSLs originate from amino acid metabolism (**Figure 6**). Mapping the pathways in this way is not merely illustrative; it localizes the late, branch-specific steps, RhaT1, UDP-rhamnose:rhamnosyltransferase 1 in GSL biosynthesis, and LAR/ANR in tannin biosynthesis, that the editing strategy proposed here prioritizes, visually separating them from the upstream steps shared with primary metabolism that should be avoided. Enzymes or genes at these late steps may therefore serve as candidate targets for reducing ANF levels, pending functional confirmation in moringa.

#### GSL biosynthesis pathway

In **Figure 6**, the dotted lines indicate the multi-step process, and the solid line indicates the single-step process. GSL biosynthesis involves three major steps: side-chain elongation, core structure synthesis, and side-chain modification. The blue arrow indicates the side chain elongation process, and the enzymes involved are BCAT3, branched-chain aminotransferase 3; BCAT4/BCAT6, branched-chain aminotransferase 4/branched-chain aminotransferase 6; IIL1, isopropyl malate isomerase large subunit 1; IMDH, isopropyl malate dehydrogenase; MAM, methylthioalkyl malate synthase. The pink arrow indicates core structure synthesis, which involves the enzymes CYP79, cytochrome P450 79; CYP83B1, cytochrome P450 83B1; GSTU19, glutathione S-transferase U19; SUR1, S-alkyl-thiohydroximate lyase; UGT74B1, UDP-glycosyltransferase 74B1; SOT, cytosolic sulfotransferase. The green arrow indicates the side chain modification, which involves the enzyme FMO_GS-OX_, flavin monooxygenase; RhaT1, UDP-rhamnose:rhamnosyltransferase 1. The yellow arrow indicates the sulfur assimilation, which involves the enzymes GSH1/GSH2, glutamate cysteine ligase1/glutamate cysteine ligase2; OASA1, O-acetylserine (thiol) lyase; APR1/APR3/APR4/APR5, 5’-adenylsulfate reductase 1/5’-adenylsulfate reductase 3/5’-adenylsulfate reductase 4/5’-adenylsulfate reductase 5; APS1, ATP-sulfurylase 1.

From a genome-editing perspective, the most promising targets in the GSL pathway are late, side-chain-modifying and tailoring enzymes that act downstream of core sulfur and amino acid metabolism, such as specific benzyl-GSL modification enzymes (RhaT1, UDP-rhamnose:rhamnosyltransferase 1). Editing this step may reduce glucomoringin and related benzyl GSLs and could minimize pleiotropic effects on primary nitrogen and sulfur assimilation. In contrast, early enzymes shared with primary metabolism (e.g., BCATs, sulfur assimilation enzymes) or broad stress-response regulators are less suitable for knockouts because their disruption is more likely to compromise growth and stress tolerance. In this case, promoter editing could be a possible solution, but it warrants investigation. However, when the transcript data are available, priority should be given to isoforms that are highly expressed in edible tissues (leaf lamina, young pods, seeds) but show limited expression in roots and reproductive organs, to preserve defensive functions where they are most needed.

#### Tannin biosynthesis pathway

The process begins with phenylalanine as the substrate, yielding p-coumaroyl CoA. The p-coumaroyl CoA, along with the malonyl CoA, forms chalcone, which in turn yields flavanone. The flavanone undergoes a set of steps to yield condensed tannins. The enzymes listed include PAL, phenylalanine ammonia-lyase; C4H, cinnamate 4-hydroxylase; 4CL, 4-coumarate-CoA ligase; CHS, chalcone synthase; CHI, chalcone isomerase; F3H, flavonoid 3-hydroxylase; F3’5’H, flavonoid 3,5-hydroxylase; DFR, dihydroflavonol reductase; LDOX, leucoantho cyanidin dioxygenase; LAR, leuco anthocyanidin reductase; ANR, anthocyanidin reductase (**Figure 7**).

In the tannin pathway, late-branch enzymes directing flux specifically to condensed tannins, such as *LAR* and *ANR*, represent better targets than the upstream phenylpropanoid enzymes (*PAL*, *C4H*, *4CL*) that also feed lignin, flavonols and other essential phenylpropanoids. Partial downregulation of these late steps, for example, through promoter editing rather than complete gene knockout, could lower tannin accumulation in leaves and seeds while potentially retaining flavonoid-based defense. Expression data can further refine target choice by highlighting isoforms that are strongly expressed in edible tissues but weak or absent in structural or reproductive organs, thereby reducing risks to plant fitness.

It should be emphasized that these candidate targets are derived from pathway homology and expression inference rather than functional validation. To date, no ANF biosynthetic gene has been knocked out or down-regulated in moringa, so the predicted effects on metabolite levels, plant defense, and fitness remain hypotheses that require experimental confirmation. The strategies proposed here are therefore best regarded as a prioritized research agenda rather than established routes to low-ANF cultivars.

Together, these omics and pathway insights suggest that genome editing in moringa should focus on tissue-specific, late biosynthetic steps with limited connections to primary metabolism and use promoter editing strategies rather than full knockouts to limit detrimental effects. On this basis, genome editing has the potential to improve moringa’s nutritional value by reducing ANFs and easing processing burdens; however, these benefits remain to be demonstrated experimentally in moringa.

### Genome editing for ANF reduction: opportunities and bottlenecks

The principal bottleneck for applying RNP-based editing in moringa is the lack of an efficient protoplast-to-plant regeneration protocol, which is essential for recovering whole edited plants from edited cells. Existing moringa tissue culture systems rely primarily on nodal explants and micropropagation, and attempts to regenerate plants from mesophyll protoplasts have not yet yielded robust, reproducible protocols. In contrast, several *Brassicaceae* crops and woody plants, including grapevine, now have highly efficient protoplast regeneration and RNP-editing pipelines (Li et al., 2021; Sandgrind et al., 2021; Tricoli and Debernardi, 2023; Le et al., 2025; Li et al., 2025; Moses et al., 2025). These successes are directly relevant to moringa, a woody species with similar recalcitrance to regeneration that shares key ANF pathways, such as GSL and phenylpropanoid biosynthesis. These parallels suggest that moringa protoplast systems could be established by systematically optimizing auxin–cytokinin ratios, osmotic conditions and culture duration across defined developmental stages, but this will require targeted, multi-year research investment.

Given these constraints, RNP-mediated editing in moringa should currently be viewed as a long-term goal rather than an immediately deployable technology. Overcoming the regeneration hurdle may realistically require several years of focused protocol development and genotype screening for this woody, perennial species. In the interim, CRISPR/Cas systems delivered by *Agrobacterium* or other vectors remain the most practical route for proof-of-concept editing, despite leaving transgenic footprints that complicate public acceptance and regulatory approval.

Grafting-based editing (described above; **Figure 5**) offers a more immediately realistic transgene-free alternative, as it sidesteps the protoplast barrier entirely by moving mobile Cas9–gRNA–TLS complexes from a transformed rootstock into a wild-type scion. Proof-of-concept has been demonstrated in *Arabidopsis* and *Brassica* species, and the approach is conceptually attractive for tree crops because the same edited scion genotype can be grafted onto a range of compatible rootstocks (Yang et al., 2023). Moringa is already propagated by cuttings and grafting for horticultural purposes, and micropropagation studies indicate that it can form functional graft unions. Still, no dedicated “editing rootstock” has yet been developed. Critical questions, therefore, include whether a readily transformable species (e.g., a related *Brassicaceae* or moringa genotype with good *in vitro* response) can serve as a graft-compatible rootstock, and how stable and efficient long-distance movement of editing transcripts would be in a woody perennial canopy. Until such work is done, graft-based editing for moringa should be considered a promising but still experimental strategy rather than a near-term breeding tool.

From a regulatory perspective, the status of genome-edited crops, including those obtained via RNP delivery or graft-mediated mobile transcripts, depends heavily on national policies for New Genomic Techniques (NGTs). Several jurisdictions now treat certain genome-edited plants that do not contain foreign DNA differently from conventional GMOs. In contrast, others still apply full GMO legislation, and debates are ongoing, particularly in the European Union (Ricroch et al., 2026). RNP-mediated and grafting-based systems that produce transgene-free seeds or scions may fall into more permissive NGT categories, but their acceptance will still depend on risk assessment and public perception of genome editing. For moringa, which is often promoted as a “natural” health food, transparent communication about editing targets (e.g., reducing specific ANFs while preserving beneficial phytochemicals) and clear separation between edited and non-edited value chains will be essential to build consumer trust.

## Comparative analysis and research priorities

**Figure 8:**
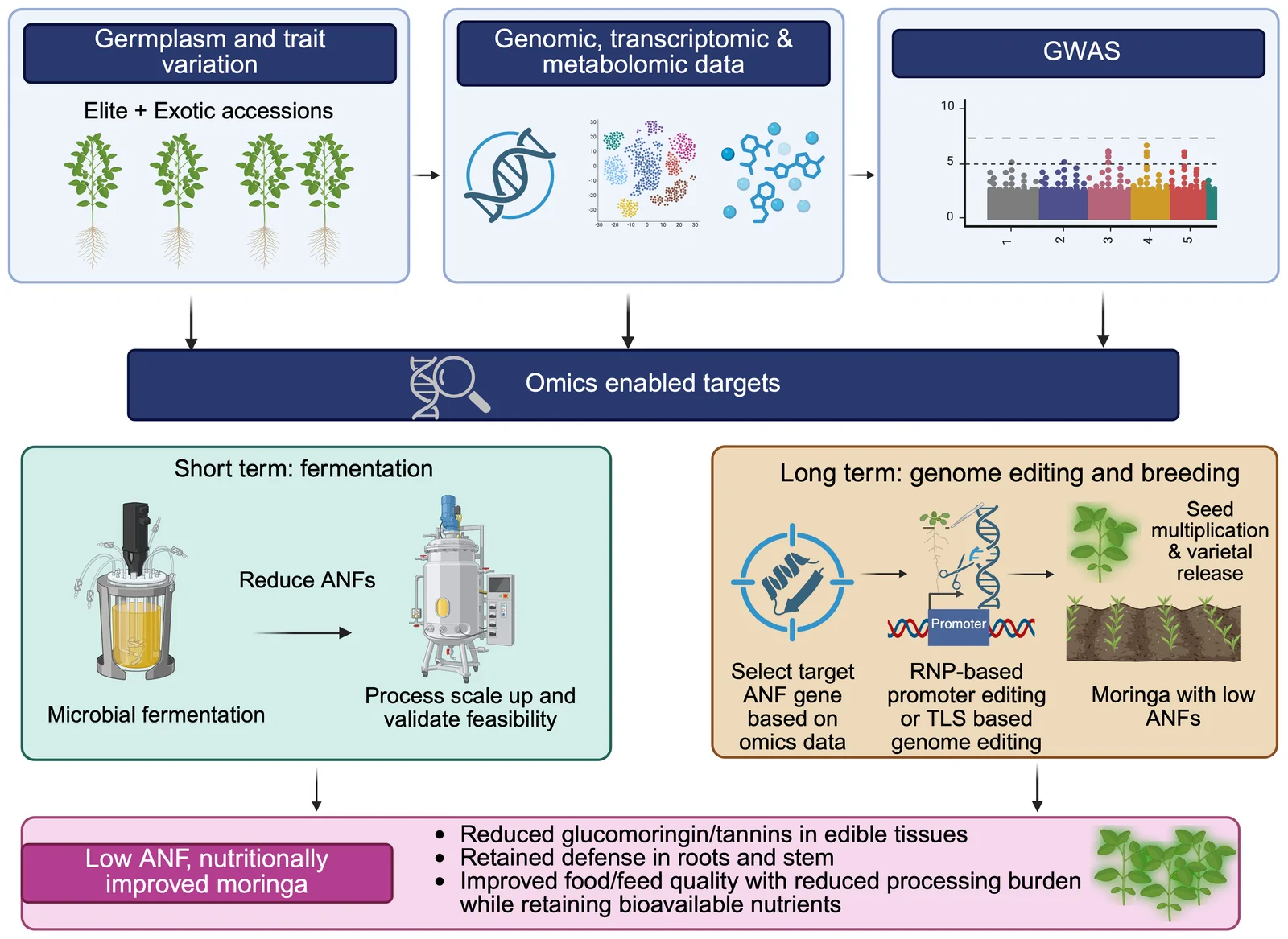
Integration of conventional breeding, fermentation, and genome-editing approaches: a roadmap for developing low-ANF, nutrient-dense *M. oleifera*. Created in BioRender. Kanagarajan, S. (2026) https://BioRender.com/9949o71.

Based on the available evidence, the three main strategies for reducing ANFs in moringa, traditional processing, microbial fermentation and genome editing, differ markedly in mechanisms, nutritional impact and timelines (Devisetti et al., 2016; Duraiswamy et al., 2023; Starzynska-Janiszewska et al., 2022). Traditional thermal or soaking methods provide moderate ANF reduction but often at the cost of vitamin, mineral and phenolic losses, whereas microbial fermentation achieves greater and more specific decreases in tannins, phytates and related compounds while enhancing bioactive components and digestibility (Ademiluyi et al., 2018; Matthew et al., 2022; Saa et al., 2022; Li et al., 2024; Perveen et al., 2024; Puspitasari et al., 2024). Genome editing offers the possibility of permanently lowering or fine-tuning ANF levels at the genetic level without compromising desirable nutrients (Confalonieri et al., 2021; Sahu et al., 2024; Menelih et al., 2025). Still, its deployment depends on robust genomic resources, tissue culture regeneration, and enabling regulatory frameworks (Bekalu et al., 2023; Cardi et al., 2023; Sebiani-Calvo et al., 2024; Bono, 2025; Fernandez Rios et al., 2025; Lokya et al., 2025; Saha et al., 2025). Among the different methods used to reduce ANFs in moringa, traditional processing methods such as soaking, heating, drying, and germination are the most economically feasible owing to their low cost and simple procedures, although they provide only moderate efficiency. Microbial fermentation is more effective in reducing compounds such as tannins and phytates and in enhancing nutrient bioavailability; however, it requires controlled conditions and additional time. In contrast, genome editing offers a highly precise and durable solution for ANF reduction at the genetic level but remains limited by high cost, technical complexity, and regulatory constraints. These contrasts are summarized in **Table 6**, which compares key features, advantages, and limitations of each approach in the context of moringa improvement. The comparison in **Table 6** reveals a consistent trade-off structure across the three approaches: cost and accessibility decline, while specificity, permanence, and technical demand rise as one moves from traditional processing through fermentation to genome editing. This gradient is what underpins our framing of fermentation and editing as complementary near- and long-term strategies rather than competing alternatives. **Figure 8** integrates these elements into a single roadmap, situating the two ANF-reduction strategies within the broader arc from germplasm and omics resources, including the GWAS, genomic selection, and systems biology capabilities that remain to be established, through candidate target identification to the shared goal of a low-ANF, nutritionally improved crop. Positioning fermentation and genome editing on a common timeline clarifies that near-term processing gains and long-term genetic solutions are sequential and mutually reinforcing rather than alternatives, with omics resources serving as the enabling layer common to both.

**Table 6:** Comparison of traditional processing and fermentation and genome editing methods of anti-nutritional factors (ANFs) reduction in *M. oleifera*.

	Traditional processing (soaking, heating, drying, germination)	Microbial fermentation	Genome editing
Main mechanisms	Leaching, thermal degradation, structural changes of ANF-containing compounds (Devisetti et al., 2016; Razzak et al., 2022)	Enzymatic degradation, acidification, and microbial metabolism by bacteria and fungi (Thierry et al., 2013; Starzyńska-Janiszewska et al., 2022).	Targeted modification or down-regulation of genes in ANF biosynthetic or regulatory pathways (Duraiswamy et al., 2023; Kumar et al., 2022).
Typical ANF reduction in moringa or similar matrices	Moderate (often 20–60%), but with significant losses of vitamins, phenolics, and minerals (Devisetti et al., 2016; Ademiluyi et al., 2018).	Higher and more specific; studies report substantial reductions in tannins, phytates and other ANFs while improving functional components (Thierry et al., 2013; Starzyńska-Janiszewska et al., 2022; Huang et al., 2025).	Potentially near-complete for selected ANFs at the gene level; effect depends on target gene and editing efficiency (Singh et al., 2020; Javaid and Choi, 2021).
Effect on nutritional quality	May decrease antioxidant activity and micronutrient content due to heat and leaching (Devisetti et al., 2016; Ademiluyi et al., 2018).	Often increases amino acid availability, bioactive phenolics, and antioxidant capacity (He et al., 2020; Starzyńska-Janiszewska et al., 2022).	Preserves native nutrient composition; changes mainly confined to targeted metabolic pathways (Ku and Ha, 2020; Kumar et al., 2022).
Infrastructure and cost	Low cost, widely accessible technologies suitable for household and small-scale processing (Ademiluyi et al., 2018; Petroski and Minich, 2020; Wickramasinghe et al., 2020; Mahamane et al., 2025).	Low–moderate cost; requires starter cultures, basic fermentation control, and food-grade facilities (Shi et al., 2020; Delfín-Portela et al., 2024; Maleke et al., 2024).	High initial R&D cost and technical capacity (genomics, transformation, tissue culture), but low marginal cost once cultivars are developed (Ahmad et al., 2021; Kalaitzandonakes et al., 2023).
Time to implementation	Immediate; can be applied to existing moringa products (Devisetti et al., 2016; Ali et al., 2017; Gharsallah et al., 2023).	Short-term; processes can be optimized and scaled within existing value chains (Zhang et al., 2017b; Wang et al., 2019; Perveen et al., 2024).	Long-term; depends on availability of annotated genomes, regeneration protocols, and regulatory approval (Cardi et al., 2017; Van Eck, 2020; Sebiani-Calvo et al., 2024; Ricroch et al., 2026).
Regulatory and social acceptance	No specific regulatory barriers; high consumer acceptance (Ntila et al., 2019; Handayani et al., 2022; Rodrigues et al., 2023).	Minimal regulatory hurdles: fermented foods are generally well accepted (Chai and Chen, 2025; Nascimento and Barros, 2025).	Strongly dependent on national policies for new genomic techniques and on public perception of genome-edited crops (Kato-Nitta et al., 2019; Spok et al., 2022; Sebiani-Calvo et al., 2024; Ricroch et al., 2026).
Sustainability and scalability	High scalability but may be energy-intensive (thermal processing) and can waste nutrients (Ali et al., 2017; Ademiluyi et al., 2018; Hegde et al., 2025).	High scalability with relatively low energy input; compatible with small- and large-scale operations (Jahanian et al., 2024; Zhai et al., 2024; Kushwaha et al., 2025).	High long-term sustainability: once developed, low-ANF cultivars reduce processing needs across production systems (Ku and Ha, 2020; Duraiswamy et al., 2023; Dellino et al., 2025; Haider et al., 2026).

## Research gaps and future thrust

Moringa is a versatile tree with numerous applications, but it still requires focused, thorough research to realize its full potential. A key goal is to conserve germplasm to protect endangered and underrepresented moringa species, avoid duplicative records, and ensure precise documentation of natural variation through detailed passport and characterization data. Creating an international central agency or network dedicated to moringa would help coordinate these efforts across countries and facilitate the sharing of germplasm and information for breeding and basic research.

Breeding efforts must more systematically focus on pod and seed traits, as well as vegetative biomass traits, that influence consumer acceptance and nutritional quality. Key priorities for pods and seeds include increased pulp content, larger seed size for table use, higher oil content, uniform pulp distribution, attractive shiny green pod color, extended shelf life, and reduced bitterness. For biomass, important goals are lower leaf and midrib bitterness, thinner petioles, minimal leaf withering during storage, enhanced iron bioavailability, strong ratooning ability, frost tolerance, rapid regrowth after pruning, and improved resistance to pests and diseases.

In parallel, there is great potential to increase moringa’s value as fodder through research on improving the nutritional quality of leaves and pods, studying the impacts of feeding on animal health and productivity, and developing value-added fodder products and optimized feed mixtures. Trials testing different feeding times and stages, as well as combinations with other forages and concentrates, would offer evidence-based recommendations for livestock systems in diverse agro-ecologies.

At the molecular level, the systematic application of advanced omics and genome-editing technologies remains in its infancy for moringa. Future work should integrate genomics, transcriptomics, metabolomics, and genome editing to examine the genetic control of ANFs, nutritional traits, and stress tolerance and to develop markers and edited lines for inclusion in breeding programs. To translate these molecular priorities into actionable research, future studies should address several testable questions. First, which loci and genes govern GSL, tannin, and phytate accumulation in moringa, and what allelic diversity is present across available germplasm? Second, what is the heritability of ANF content in seeds and leaves, and how are these traits genetically correlated with yield and nutritional quality? Third, among the homology-based candidates listed in **Table 4**, which are expressed in the tissues where the respective ANFs accumulate, and which are likely to function in pathway-specific rather than primary-metabolism processes? Fourth, do targeted edits that reduce a given ANF produce measurable effects on plant defense, fitness, or beneficial phytochemical composition? Finally, can microbial fermentation be standardized across strains, substrates, and incubation durations and scaled to deliver reproducible, food-grade reductions in ANF content? Collectively, answering these questions would move the field from broad aspiration toward a well-defined experimental roadmap. Furthermore, research on human health–related products, such as nutrient tablets and functional health products, along with the design of sustainable, efficient value chains and marketing strategies, is necessary to translate agronomic and molecular advancements into accessible moringa-based foods and supplements.

Finally, supportive policy and institutional frameworks will be essential for expanding the improvement and use of moringa. Key actions include recognizing moringa as a strategic crop in both national and international research agendas, establishing dedicated crop improvement programs, and developing regulations that support products derived from advanced breeding and genome editing. Coordinated investments by government and donors in research, extension services, and processing infrastructure will be vital to realizing moringa’s full scientific and economic potential.

## Conclusion

Moringa is a multipurpose tree crop that offers significant scope for nutritional sustainability. A suite of ANFs, notably phytic acid, tannins, GSLs, saponins, and lectins, reduces its consumption. This review shows that (i) the moringa germplasm characterization is in its primitive stage, warrants establishment of a global nodal agency, (ii) classical hybridization methods were mostly utilized for annual moringa improvement, but intensive genetic improvement work in the perennial types are to be initiated/intensified, and (ii) microbial fermentation and genome editing provide complementary pathways to manage the ANF compounds rather than competing alternatives. In the near term, optimized lactic acid and solid-state fermentations with carefully selected bacterial, fungal, and yeast consortia can achieve 50–70% reductions in phytate, tannins, and related GSLs while simultaneously improving mineral bioavailability, amino acid profiles, and sensory quality in moringa-based products. Over the long term, CRISPR-based genome editing, particularly promoter editing and transgene-free approaches, offers the potential to durably reduce ANF accumulation directly in the plant without compromising desirable phytochemicals or agronomic performance.

Our pathway-level synthesis highlights specific late biosynthetic steps as particularly promising targets, including tailoring enzymes in GSL biosynthesis and branch-point enzymes such as LAR and ANR in condensed tannin formation, which can be modulated to reduce ANFs while preserving core phenylpropanoid and sulfur metabolism. As noted above, deployment remains constrained chiefly by incomplete genome annotation and the absence of reproducible protoplast regeneration systems. Addressing these bottlenecks, alongside continued investment in fermentation optimization, product development, and regulatory frameworks, will be key to moving from proof-of-concept studies to scalable impact. The integrated roadmap in **Figure 8** summarizes this trajectory, linking germplasm and omics foundations through candidate target identification to the complementary near-term fermentation and long-term editing strategies that together can deliver low-ANF moringa. By integrating traditional fermentation knowledge with cutting-edge genome editing, moringa can transition from an underutilized “miracle tree” to a mainstream nutritional powerhouse that contributes meaningfully to global food and nutrition security.
